# SOD1 protein aggregates stimulate macropinocytosis in neurons to facilitate their propagation

**DOI:** 10.1186/s13024-015-0053-4

**Published:** 2015-10-31

**Authors:** Rafaa Zeineddine, Jay F. Pundavela, Lisa Corcoran, Elise M. Stewart, Dzung Do-Ha, Monique Bax, Gilles Guillemin, Kara L. Vine, Danny M. Hatters, Heath Ecroyd, Christopher M. Dobson, Bradley J. Turner, Lezanne Ooi, Mark R. Wilson, Neil R. Cashman, Justin J. Yerbury

**Affiliations:** Illawarra Health and Medical Research Institute, Wollongong, Australia 2522; School of Biological Sciences, Faculty of Science, Medicine and Health, University of Wollongong, Wollongong, Australia 2522; Intelligent Polymer Research Institute, University of Wollongong, Wollongong, Australia 2522; Australian School for Advanced Medicine, Macquarie University, Sydney, Australia 2109; Department of Biochemistry and Molecular Biology and Bio21 Molecular Science and Biotechnology Institute, University of Melbourne, Parkville, Australia 3010; Department of Chemistry, University of Cambridge, Cambridge, CB2 1EW UK; Florey Institute of Neuroscience and Mental Health, University of Melbourne, Parkville, Australia 3010; Department of Medicine (Neurology), University of British Columbia and Vancouver Coastal Health Research Institute, Brain Research Centre, University of British Columbia, Vancouver, Canada V6T 2B5

**Keywords:** Protein aggregation, Transmission, Macropinocytosis

## Abstract

**Background:**

Amyotrophic Lateral Sclerosis is characterized by a focal onset of symptoms followed by a progressive spread of pathology that has been likened to transmission of infectious prions. Cell-to-cell transmission of SOD1 protein aggregates is dependent on fluid-phase endocytosis pathways, although the precise molecular mechanisms remain to be elucidated.

**Results:**

We demonstrate in this paper that SOD1 aggregates interact with the cell surface triggering activation of Rac1 and subsequent membrane ruffling permitting aggregate uptake via stimulated macropinocytosis. In addition, other protein aggregates, including those associated with neurodegenerative diseases (TDP-43, Htt_ex1_46Q, *α*-synuclein) also trigger membrane ruffling to gain entry into the cell. Aggregates are able to rupture unstructured macropinosomes to enter the cytosol allowing propagation of aggregation to proceed.

**Conclusion:**

Thus, we conclude that in addition to basic proteostasis mechanisms, pathways involved in the activation of macropinocytosis are key determinants in the spread of pathology in these misfolding diseases.

**Electronic supplementary material:**

The online version of this article (doi:10.1186/s13024-015-0053-4) contains supplementary material, which is available to authorized users.

## Background

The hallmark of Amyotrophic Lateral Sclerosis (ALS) is the selective death of upper and lower motor neurons in the motor cortex, brainstem and spinal cord, leading to loss of voluntary muscle control, muscle atrophy and invariably death. The specific causes of most cases of ALS are undefined, although approximately 10 % are inherited. The best-studied familial ALS (fALS) cases are from families possessing mutations in the gene encoding copper/zinc superoxide dismutase (Cu/Zn SOD, *SOD1*) [1]. There is, however, a rapidly growing list of other genes in which mutations have been implicated in fALS. These include *ALS2*, *SETX*, *FUS*, *VAPB*, *ANG*, *TARDBP*, *OPTN*, *VCP*, *UBQLN2*, *PFN1, SQSTM1*, and a hexanucleotide repeat in a non-coding region of *C9ORF72 *[2]. Current clinical practices are such that by the time that a diagnosis is confirmed disease progression is well under way and as many as 50 % of motor units may already have been affected [3]. There is now very strong evidence in humans that neurodegeneration in ALS begins focally and then spreads amongst adjacent motor neurons or through axonal pathways throughout the three dimensional anatomy of the central nervous system [4–6]. More detailed knowledge of the action of this spreading is crucial, as is identifying a means of early detection of the disease if we are to therapeutically slow disease progression.

In common with other neurodegenerative diseases, such as Alzheimer’s Disease and Parkinson’s Disease [7, 8], there is growing evidence that disruptions to proteostasis, protein misfolding and aggregation are the underlying mechanisms driving neurodegeneration in ALS [9]. Of particular interest is the fact that, although nucleation of protein aggregation appears to be a stochastic process [10], suggesting that protein aggregation should cause a random pattern of cell death, cell death in ALS occurs in an ordered and progressive manner. One way to explain such ordered progression is the prion-like propagation of protein misfolding and aggregation between adjacent cells. In addition, recent work has indicated that secondary processes, notably nucleation [11–13], takes place on the surface of aggregates, and along with other diffusional or active transport of protein aggregates between cells can give rise to cell-to-cell propagation of the type that is often defined as prion-like behaviour.

In the specific context of ALS, previous work has shown that exogenously applied mutant SOD1 aggregates induce protein aggregation in cells overexpressing mutant SOD1 [14]. Importantly, recent work shows that injection of spinal cord homogenates from symptomatic G93A SOD1 mice into the sciatic nerve of mice expressing G85R SOD1-YFP, below the threshold for triggering disease, has been shown to kindle protein aggregation and subsequent ALS-like phenotype [15]. In addition, we have recently shown, using cell lines and primary neurons, that propagation of SOD1 misfolding is dependent upon the passage of misfolded SOD1 (either mutant or wt) from cell-to-cell, a process that can be neutralized by antibodies reactive with misfolded SOD1 epitopes [16]. We have also shown that soluble and aggregated SOD1 can be taken up by neuroblastoma cells (mouse NSC-34 and human SHSY5Y), after which it accumulates in cytosolic inclusions [17]. Clues to understanding the entry of aggregates into cells comes from studies that show uptake of aggregates of SOD1 can be blocked with EIPA, wortmannin, IPA-3 [14] and rottlerin [16, 17], which inhibit Na^+^/H^+^ exchangers, phosphoinositide 3-kinases (PI3K), P21 protein (Cdc42/Rac)-activated kinase 1 (PAK-1) and protein kinase C (PKC), respectively (suppressing signalling events that promote actin rearrangement and pinosome closure). These findings suggest the involvement of fluid phase pinocytosis, possibly macropinocytosis, in the aggregate uptake process. Similarly, we have shown that cellular uptake of soluble SOD1 can be blocked by co-treatment with EIPA or rottlerin also suggesting that a form of pinocytosis plays a role in this process [17].

Macropinocytosis is a form of non-selective endocytosis used by cells to engulf large amounts of solute macromolecules (fluid phase) or particles too large for other forms of endocytosis. Macropinocytosis is typically defined as a transient, externally induced, actin-dependent endocytic process associated with vigorous perturbations, such as ruffles and blebs, in the plasma membrane [18]. The activation of this process results in a transient increase in receptor-independent fluid phase endocytosis in large vesicles or vacuoles (0.5–10 μm) termed macropinosomes [18, 19]. Macropinosomes do not have a coat to guide their formation and are heterogeneous in size and shape. Macropinocytosis provides non-phagocytic cells with the ability to take up large particles, but the process must be triggered by an external stimulus. Bacteria, viruses, apoptotic bodies and necrotic cells have all been shown to induce the ruffling behaviour typical of macropinocytosis resulting in their uptake along with fluid [18].

There are a number of viruses, including the vaccinia virus and adenovirus [20], that have been shown to hijack macropinocytosis pathways to enter cells, including the Japanese encephalitis virus that triggers macropinocytosis in neurons [21]. A lack of physical structure is thought to result in the loss of integrity of the macropinosome membrane and may explain why particles (e.g. bacteria and virions) can escape the macropinosomes and reach the cytosol [22]. There is emerging evidence that prions and prion-like proteins may also enter cells via macropinocytosis allowing the propagation of their aggregation [14, 16, 17, 23, 24]. However, the small molecule inhibitors utilized to define macropinocytosis, such as EIPA, are not specific to a single cellular event and depending on the cell type can prevent various forms of endocytosis [25].

We show here however, that aggregates of SOD1 trigger activation of the Rho GTPase Rac1, leading to membrane ruffling and fluid phase uptake in neurons that defines macropinocytosis and that this facilitates aggregate entry into cells. In addition, we show that these aggregates can escape macropinosomes to be deposited in the cytosol. We further show that this process is not specific for SOD1 but rather can be triggered by a variety of protein aggregates, including model protein aggregates (α-lactalbumin) and those associated with neurodegenerative diseases (TDP-43, Htt_ex1_46Q and α-synuclein). We conclude that the infectious prion-like spread of protein aggregation in a range of neurodegenerative diseases is dependent on cells activating ruffling and subsequent macropinocytosis in a manner analogous to viral entry and propagation through activation of macropinocytosis.

## Results

### SOD1 aggregates are taken up by cells and promote wtSOD1 inclusion formation

**Fig. 1:**
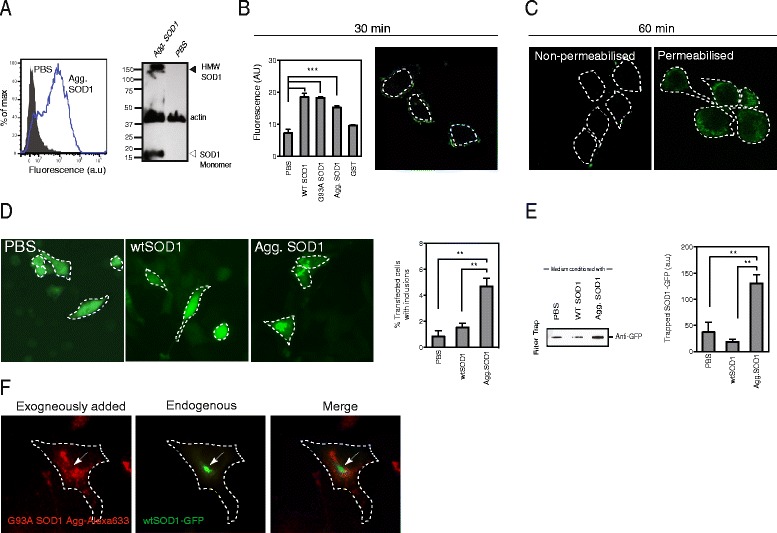
Exogenously applied SOD1 aggregates enter cells and induce endogenous SOD1 aggregation. **a** *Left panel* Quantitative analysis of SOD1 association with NSC-34 cells using flow cytometry. Cells were either incubated with PBS (grey) or aggregated wtSOD1 (60 min incubation; blue line). *Right panel* Western blot of cell lysates detecting human SOD1 (and actin as a loading control). (**b**) *Left panel* Association with cells was quantified using flow cytometry. NSC-34 cells were treated with aggregated human SOD1 protein for 30 min and subsequently detected using immunofluorescence. Results shown are means ± SE, *n* = 3, ** *p* < 0.01. *Right panel* Confocal laser scanning micrograph of aggregated  wtSOD1 interacting with NSC34 cells after 30 min on ice to slow endocytosis. White dotted line represents cell membrane. (**c**) Confocal laser scanning micrographs of biotinylated wtSOD1 aggregates incubated with NSC34 cells for 60 min then either permeabilized with Triton x-100 or not, and subsequent detection using SA-Alexa488. (**d**) NSC-34 cells were transfected with wtSOD1-GFP and then incubated with either PBS, wtSOD1 (non-aggregated) or aggregated SOD1 and the number of cells with inclusions counted at 72 h. Results shown are means ± SE, *n* = 3, * *p* < 0.05. A minimum of 150 cells were counted per treatment and the average % of transfected cells with inclusions calculated across a minimum of 5 fields of view per treatment. (**e**) Cell lysates were further analyzed by filter trap assay. Any trapped SOD1-GFP material was measured using an anti-GFP antibody to avoid measuring aggregated material added to cells. Quantification of filter trap assays using a densitometer. Values are the mean intensity of trapped aggregated material averaged over 3 experiments. Results shown are means ± SE, *n* = 3, ** *p* < 0.01. (**f**) Exogenously added and endogenously produced SOD1 aggregates do not substantively colocalise. G93A SOD1 aggregates were labelled with Alexa-633 and added to NSC-34 cells expressing wtSOD1-GFP. After 48 h the cells were imaged using laser scanning confocal microscopy. Little colocalisation of Alexa633 and GFP signal was observed

We, and others, have previously shown that aggregated human SOD1 can be taken up by neuronal cells [14, 16, 17]. Here we confirm these results using recombinantly produced properly folded and dimeric human SOD1 that was aggregated in vitro to form fibrillar structures, as previously described [26] (Additional file 1A-B). The preformed SOD1 aggregates were then added to the media of the motor neuron like cell line NSC-34. Following incubation of the cells with the SOD1 aggregates for 60 min, the aggregates could be detected in association with the cells by flow cytometry (Fig. 1a). In addition, human SOD1, including SDS resistant high molecular weight (HMW) species, was detected in lysates from trypsin treated cells (Fig. 1a). Furthermore, to eliminate the possibility that SOD1 aggregates were bound primarily to the cell surface we treated cells with trypsin and observed no difference in SOD1 signal consistent with SOD1 being inside cells (Additional file 2). Putative uptake of SOD1 was quantified by flow cytometry and it was found that soluble (properly folded) wtSOD1 and non-aggregated ALS associated mutant G93A SOD1 are taken up by cells to a similar extent to that of aggregated wtSOD1 (Fig. 1b). This is consistent with previous work showing uptake of soluble wt and mutant SOD1 [17] and may reflect the proposed role of non-classically secreted SOD1 in signal transduction [27, 28]. In contrast, there was no statistically significant increase in immunofluorescence after incubation with an unrelated control protein GST, suggesting that the uptake of both soluble and aggregated forms of SOD1 is relatively specific (Fig. 1b). The uptake of aggregates is dependent upon cell surface proteins, since trypsinization of cell surface proteins prior to treatment with aggregates significantly inhibited aggregate association with cells (Additional file 1C). In addition, uptake occurs relatively rapidly; SOD1 aggregates were associated with the surface of NSC-34 cells after 30 min incubation (Fig. 1b), and after 60 min are no longer detected on the surface but instead are only detected following permeabilisation of cells with Triton X-100 (Fig. 1c).

Previous work has shown that exogenously added preformed aggregates of mutant SOD1 containing fibrils can induce aggregation of intracellular SOD1 in cells overexpressing mutant SOD1 [14], but not in those expressing wtSOD1. Recently we have shown that wtSOD1 can indeed participate in the propagation of misfolded SOD1 within and between cells [16, 29]. To further examine the induced aggregation of intracellular wtSOD1, we transfected NSC-34 cells with wtSOD1-GFP and added soluble or aggregated recombinant SOD1 to the media. After 48 h of incubation there were more cells that contained wtSOD1-GFP inclusions when treated with aggregated SOD1 than when treated with soluble SOD1 (Fig. 1d). As the exogenously added aggregates were not  labelled with GFP these cellular inclusions could not be attributed to the uptake of aggregates but must have formed from intracellular wtSOD1-GFP. The number of cells expressing wtSOD1-GFP that spontaneously developed inclusions was low (< 1 % for cells treated only with PBS) and occurred only in cells expressing very high levels of wtSOD1-GFP [29, 30]. As we did not observe substantive colocalisation of the exogenously applied SOD1 aggregates and SOD1-GFP, our results suggest accumulation of SOD1-GFP occurs alongside aggregates taken up from the media (Fig. 1f). Exogenous application of aggregated SOD1 resulted in a highly significant (*p* < 0.0001) increase (from 0.85 +/− 0.42 % to 4.7 +/− 0.62 %) in the proportion of cells containing inclusions (Fig. 1d). We confirmed these data with a filter trap assay using cell lysates (Fig. 1e). Increased levels of SOD1-GFP were trapped on the cellulose membrane when cells were treated with aggregated wtSOD1 than those treated with soluble wtSOD1 or PBS alone (Fig. 1e; *p* < 0.001). Similar results were also seen when aggregates made from several mutant SOD1 variants that cause ALS were aggregated in vitro and added to cells expressing wtSOD1-GFP (Additional file 3).

### SOD1 aggregates escape endolysosomal system to access the cytosol

**Fig. 2:**
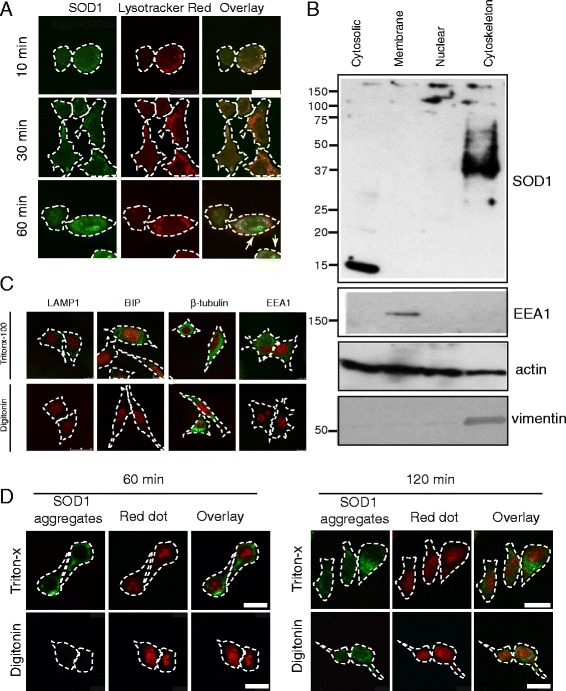
Internalized aggregates entering via the endocytic pathway escape from the endosomes to the cytosol. **a** Confocal microscopy of the co-staining of wtSOD1 aggregates (20 μg/mL) taken up by NSC-34 cells after 10, 30 and 60 min at 37 °C, with Lysotracker Red. Fixed and permeabilized cells were immunostained with an anti-human specific SOD1 antibody. Bars represent 25 μm. Arrowheads indicate areas of localisation of SOD1 outside the acidic compartment. (**b**) Western blotting of cytosolic, membrane (ER/Golgi), nuclear and cytoskeleton fractions (10 μg) collected from NSC-34 cells treated with wtSOD1 aggregates (20 μg/mL) for 2 h at 37 °C. Western blots were stained for the presence of human SOD1, EEA1, actin and vimentin. (**c**) Digitonin selectively permeabilizes plasma membrane of NSC-34 cells. NSC-34 Cells were treated with either digitonin (10 μM) or Triton-x100 (0.5 %) and then immunostained for membrane bounded markers LAMP1, BIP and EEA and the cytosolic β-tubulin. Membrane bounded markers were not detected when permeabilized with digitonin indicating the specificity of digitonin for plasma membrane. Cells were counterstained with Red Dot 2. (**d**) Laser scanning confocal micrographs of NSC-34 cells were treated with aggregated human SOD1 protein for 60 or 120 min then fixed and permeabilized with either Triton-x100 (0.5 %) or digitonin (10 μM). SOD1 aggregates were detected using Alexa488 conjugated to streptavidin and counter stained with the nuclear dye Red Dot 2. SOD1 aggregates are only detected upon digitonin permeabilization after 120 min SOD1 incubation. White dotted line represents cell membrane

The mechanism by which exogenously applied SOD1 aggregates induce the formation of cytoplasmic inclusions containing intracellular SOD1 likely involves the escape of SOD1 from membrane bound endolysosomes. To test whether SOD1 leaks from endo-lysosomal compartments following its uptake, NSC-34 cells were incubated with SOD1 aggregates at 37 °C and following incubation at incremental time intervals co-stained for SOD1 aggregates and lysosomes. Exogenous SOD1 aggregates co-localised with Lysotracker (fluorescent in mildly acidic compartments) until 30 min, after which low levels of SOD1 could be observed outside the acidic endo-lysosomal system (Fig. 2a). To determine if SOD1 aggregates had entered the cytosol we first performed sub-cellular fractionation of cells. Immunoblotting of cytosolic, membrane (ER/Golgi), nuclear and cytoskeletal fractions demonstrated that aggregated SOD1 predominantly fractionated with the cytoskeleton fraction (Fig. 2b). We observe upon boiling in SDS that the aggregates no longer appear as high molecular weight species stuck in the loading gel (see Fig. 1a), but are reduced to apparent cross-linked species as previously observed [31]. These results are consistent with SOD1 aggregates having a density comparable to cytoskeleton elements and, in addition, suggests cytosolic exposure of SOD1 aggregates. There was also a very small amount of high molecular weight SOD1 observed in the nuclear fraction, again consistent with cytosolic exposure (outside of the endo-lysosome system). However, in confocal microscopy experiments aggregates were not observed within the nucleus (Fig. 2d) suggesting aggregated material either bound the nuclear membrane or pelleted at a similar density to the nuclear fraction.

To confirm that aggregates were present outside any membrane enclosed compartments we used selective permeabilization; Triton- X-100 disrupts all cellular membranes, while digitonin permeabilizes only the plasma membrane [32] (see also control membrane enclosed [Lysosomal associated membrane protein 1; LAMP1, Binding immunoglobulin protein; BiP, Early endosome antigen 1; EEA1] and cytosolic proteins shown in Fig. 2c). After 60 min incubation, human SOD1 aggregates were detected only after Triton X-100 permeabilization (Fig. 2d). However, after 120 min, SOD1 aggregates could also be detected after permeabilization by digitonin, consistent with aggregate presence in the cytosol. Soluble human SOD1 was also able to escape the endosome system, such that it was detected in the cytosol after digitonin permeabilization (Additional file 4). In contrast, when the control protein RAP-GST (known to be internalized by receptor mediated endocytosis) was added to cells for 120 min it was not detected after digitonin permeablization (Additional file 4), consistent with its retention in an endosomal-lysosomal compartment.

**Fig. 3:**
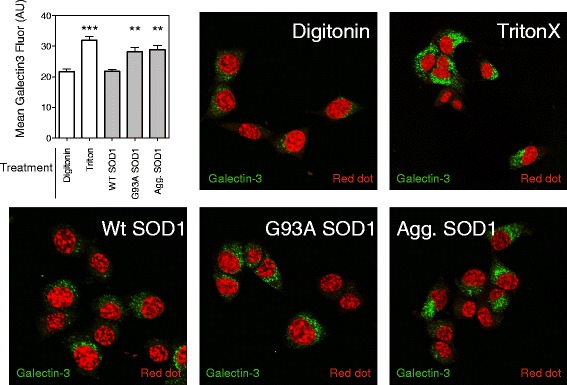
Endosome rupture by SOD1 aggregates. NSC-34 cells were incubated with either PBS (digitonin and Triton X-100 treatments), non-aggregated wt or G93A SOD1 or aggregated G93A SOD1 for 2 h before permeabilizatin (digitonin for the SOD1 treatments), fixation, immunostaining of galectin-3 and the mean fluorescence per cell quantified. A minimum of 150 cells were counted per treatment across a minimum of 5 fields of view per treatment and the mean galectin-3 fluorescence per cell calculated. Results shown are means ± SE, ** *p* < 0.01, *** *p* <0.001. Confocal microscopy of the immunostaining of galectin-3 after treatment with PBS and the SOD1 preparations, counterstained with RedDot

Macropinosomes are thought to be ‘leaky’ due to their lack of physical structure [22]. In order to test the possibility that SOD1 aggregates are rupturing macropinosomes we specifically permeabilized only the plasma membrane with digitonin and then stained for galectin-3 after 2 h of incubation with SOD1 aggregates. Galectin-3 is a non-classically secreted β-galactoside binding lectin that has been shown to be enriched in Rab positive endosomes [33] and also redistributes from the cytosol to endosome fragments upon endosome rupture [34, 35]. We utilized our selective permeabilization method (see Fig. 2c) to examine increased cytosolic exposure of galectin-3 indicating rupture of endosomal compartments. Cells permeabilized by digitonin and treated with PBS alone showed very little galectin-3 staining compared to cells permeabilized with Triton x-100 (Fig. 3), consistent with a large fraction of galectin-3 being located on the inside endosomal compartments [33] in NSC-34 cells. Compared to controls, the cultures treated with misfolded G93A SOD1 or aggregated SOD1 contained an increase in staining for galectin-3 indicative of endosome rupture and exposure of galectin-3 (Fig. 3). In addition, we treated human red blood cells with the SOD1 samples and the amount of membrane disruption was measured by free hemoglobin (Additional file 5A). Soluble mutant G93A SOD1, and aggregated SOD1 increased the amount of free hemoglobin consistent with an ability to damage biological membranes (Additional file 5A). Lastly, we generated unilamellar vesicles and examined for interaction of SOD1 aggregates. Similar to previous findings for fibrillar aggregates [36], aggregates of SOD1 interacted with liposomes and deformed their structure (Additional file 5B). Together, these data are consistent with SOD1 aggregates disrupting biological membranes and escaping in to the cytosol.

### Aggregated SOD1 triggers ruffling and subsequent macropinocytosis in neurons

**Fig. 4:**
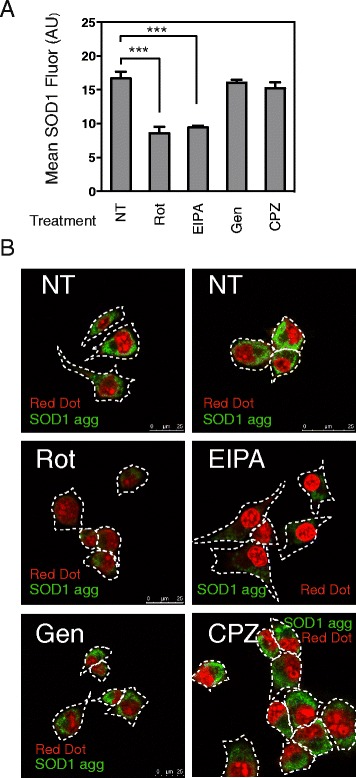
Rottlerin and EIPA treatment inhibits the uptake of SOD1 aggregates. **a-b** Internalization of aggregated wtSOD1 (20 μg/mL) after 30 min at 37 °C, in the absence (no treatment; NT) or presence of a pre-incubation step with either rottlerin (Rot; 3 μM) a PAK-1 and PKC inhibitor, EIPA (100 μM) a Na^+^/H^+^ exchange inhibitor, genistein (Gen; 10 μM) a caveolin mediated uptake inhibitor, or chlorpromazine hydrochloride (CPZ; 5 mM) a clathrin mediated endocytosis inhibitor. **a** The mean fluorescence intensity (MFI) of SOD1 aggregate uptake was determined by flow cytometry. Results shown are means ± SD, *n* =6, *** P <0.001 (**b**) Laser scanning confocal micrographs of aggregated wtSOD1 internalized by NSC-34 cells in the presence or absence of a pre-incubation step with rottlerin (Rot), EIPA, genistein (Gen) and chlorpromazine hydrochloride (CPZ)

Previous studies have shown that small molecules that inhibit actin rearrangement, or Na^+^/H^+^ exchangers, Pak-1, PI3K, and PKC suppress aggregate uptake [14, 16, 17], consistent with macropinocytosis. However, it has not been shown whether macropinocytosis is triggered through an interaction of SOD1 with cells or whether aggregates are taken up by some other constitutive process. Initially, we used EIPA (an inhibitor of the Na^+^/H^+^ exchanger and subsequent endocytosis) and rottlerin (an inhibitor of PKC), as reported previously [14, 16, 17], to confirm the involvement of macropinocytosis-like pathways in the uptake of SOD1 aggregates into NSC-34 cells. Aggregate uptake was inhibited by both EIPA and rottlerin (*p* < 0.001; Fig. 4a-b), however inhibitors of clathrin (chlorpromazine) or caveolin (genistein) dependent endocytosis had no significant effect on this process (Fig. 4a-b). Macropinocytosis is a form of fluid phase endocytosis that engulfs solutes at whatever concentrations they are found in the extracellular medium, rather than concentrating ligands at the cell surface. EIPA, cytochalasin D (an inhibitor of actin rearrangement) and rottlerin inhibited fluid phase uptake (quantified as dextran-Alexa647 uptake) stimulated by phorbol 12-myristate 13-acetate (PMA) treatment of NSC-34 cells, but genistein and chlorpromazine did not, demonstrating the specificity of the inhibitors to PKC-dependent fluid phase uptake (Additional file 6A). Similar results were found regardless of whether or not the aggregates were wt or mutant G93A SOD1 (Additional file 6B). While a similar pattern of SOD1 uptake inhibition was found when soluble non-aggregated wtSOD1 was applied to cells (largest decreases in fluorescence when co-incubated with EIPA and rottlerin; Additional file 6C), non-aggregated G93A SOD1 uptake was inhibited by similar levels regardless of the inhibitor used (Additional file 6D).

**Fig. 5:**
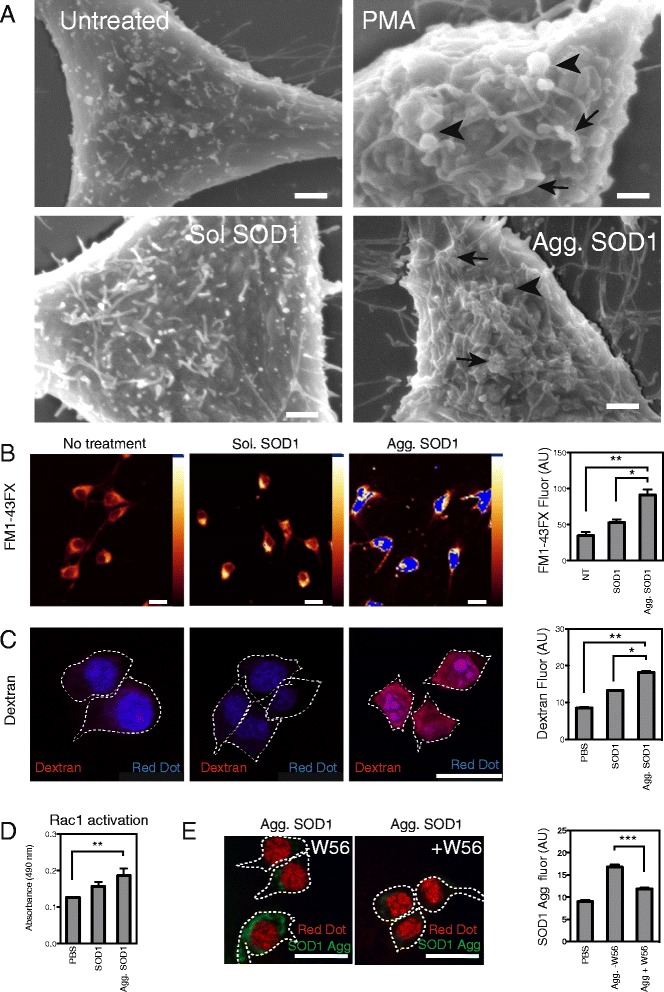
Aggregated SOD1 induces ruffles and blebs, dextran uptake and activation of RAC1. **a** Field emission SEM of cells treated with SOD1 aggregates or controls, non-aggregated SOD1 (sol SOD1), PMA or PBS (untreated). Increases in membrane perturbations can be observed, such as ruffles (black arrow) and blebs (black arrow head). Bars represent 2 μm. (**b**) Laser scanning confocal micrographs of treated cells stained with the membrane dye FM1-43FX to measure membrane perturbation and fluorescence intensity per cell was quantified using ImageJ. Scale bars represent 20 μm. A minimum of 200 cells were scored per treatment. Results shown are means ± SD of three experiments, * *p* < 0.05 ** *p* < 0.01. (**c**) The induction of fluid phase uptake was measured using fluorescently labelled dextran. Laser scanning confocal micrographs of dextran-Alexa647 uptake in treated NSC-34 cells. Outline of cells are indicated with white dashed lines. Scale bars represent 20 μm. (**e**) Flow cytometry quantification of dextran uptake in the treated NSC-34 cells. Results shown as means ± SD of 6 experiments * p <0.05 ** *p* < 0.01. (**d**) Rac1 activation in treated NSC-34 cell lysates was measured using a Rac1 activation ELISA assay that probes for Rac1-GDP**.** Results are mean ± SD of 6 experiments, ** *p* < 0.01. (**e**) Addition of a Rac1 inhibitor reduces SOD1 uptake. Laser scanning confocal micrographs of SOD1 aggregate uptake in the presence of absence of W56, mean fluorescence per cell was calculated using ImageJ. Data are mean fluorescence intensity per cell of a minimum of 100 cells ± SD, *p* < 0.001

We next investigated whether there were any perturbations to the cell surface membrane caused by incubation with SOD1. Field emission scanning electron microscopy (FESEM) imaging of cells treated with PMA showed increased membrane perturbations, including ruffles and blebs (Fig. 5a), consistent with an activation of macropinocytosis. Incubation with soluble G93A SOD1 did not induce such perturbations, although incubation with aggregated G93A SOD1 induced pronounced membrane ruffling and blebbing consistent with macropinosome formation (Fig. 5a). To exclude the possibility that cells were blebbing due to apoptosis, we examined the cells for active caspase 3, however there was no caspase 3 activation in cells treated with aggregates above basal (PBS treated) levels (Additional file 7). To visualize and quantify the extent of membrane perturbation we used the membrane dye FM® 1-43FX, as used previously for studies of membrane perturbation during growth cone ruffling [37]. Fluorescence from FM 1-43FX was significantly increased (*p* < 0.001) upon treatment of cells with SOD1 aggregates, consistent with an increase in membrane perturbation (Fig. 5b). In contrast, there was no increase in fluorescence following incubation with soluble SOD1 (Fig. 5b).

Next, to explore whether the interaction of SOD1 aggregates with cells triggers fluid phase uptake, we incubated NSC-34 cells in the presence of both SOD1 and dextran-Alexa647 and quantified uptake of the latter using flow cytometry. Aggregated SOD1 triggered a significant increase (*p* < 0.001) in dextran uptake compared to that in the absence of treatment or following incubation with soluble SOD1 (Fig. 5c). Whilst there was a small increase in dextran uptake in cells treated with soluble SOD1 compared to those not treated (Fig. 5c) this was thus not attributable to stimulated macropinocytosis as it occurred in the absence of membrane ruffling (Fig. 5a). Finally, we examined the role of the Rho GTPase, Rac1, in the uptake of SOD1 aggregates. Initially we probed for the presence of activated Rac1 using an ELISA based assay. Incubation of cells with aggregated but not with soluble SOD1 resulted in a significant increase (*p* < 0.05) in the amount of activated Rac1 (Rac1-GTP; Fig. 5d) in NSC-34 cells. In addition, the uptake of SOD1 aggregates was significantly suppressed (*p* < 0.001) by pre-treatment and subsequent co-incubation of aggregates with the Rac1 inhibitor W56 (Fig. 5e). To confirm that Rac1 was downstream of membrane ruffling we treated cells with PMA or SOD1 aggregates in the presence or absence of W56 and then examined for membrane perturbations using FM1-43FX. We observed that in the case of both PMA and aggregate treated cells W56 suppressed membrane perturbation (Additional file 8).

**Fig. 6:**
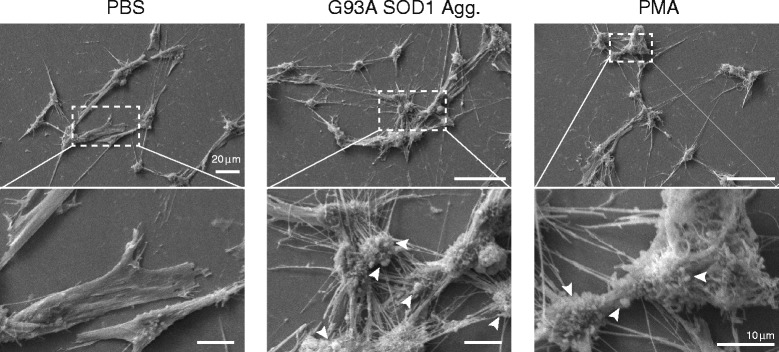
Aggregated SOD1 induces membrane ruffling in iPSC derived human motor neurons. Field emission SEM of motor neurons treated with SOD1 aggregates, and PBS or PMA controls. The area in the dashed box is enlarged in the bottom panels. Increases in membrane perturbations can be observed, such as ruffles and blebs (white arrow heads). Bars represent 20 μm in top row and 10 μm in the bottom row. Data shown is from experiments performed on cells derived from one fibroblast line and represents experiments performed on cells from 2 individuals

**Fig. 7:**
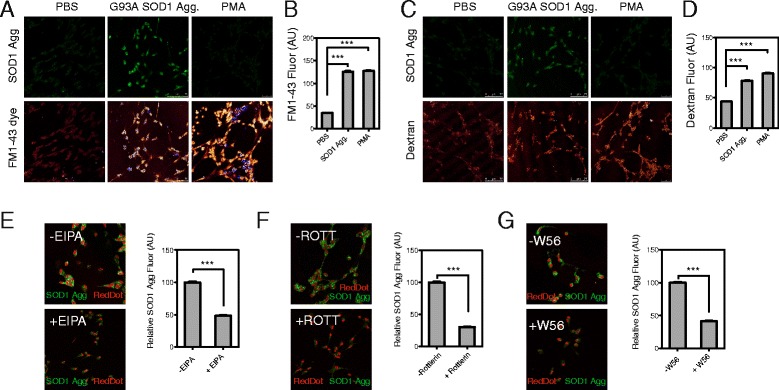
SOD1 aggregates activate membrane perturbation and dextran uptake in iPSC derived human motor neurons. Laser scanning confocal micrographs of treated cells stained with the membrane dye FM1-43FX (**a**) to measure membrane perturbation and fluorescence intensity quantification using ImageJ (**b**). A minimum of 100 cells were scored per treatment. Results shown are means ± SD of three experiments, * *p* < 0.05 ** *p* < 0.01. The induction of fluid phase uptake was measured using fluorescently labelled dextran. (**c**) Laser scanning confocal micrographs of dextran-Alexa647 uptake in treated motor neurons. (**d**) ImageJ quantification of dextran uptake in the treated motor neurons. A minimum of 100 cells per treatment were scored. Results shown as means ± SD of 3 experiments * p <0.05 ** *p* < 0.01. (**e**) Laser scanning confocal micrographs of aggregated SOD1 internalized by motor neurons in the presence or absence of a pre-incubation step with macropinocytosis inhibitors EIPA (**e**), Rottlerin (**f**) and rac1 inhibitor W56 (**g**). Fluorescence was quantified by imageJ. Data are mean fluorescence intensity per cell of a minimum of 100 cells ± SD, *p* < 0.001. Data shown is from experiments performed on cells derived from one fibroblast line and represents experiments performed on cells from 2 individuals

We also used human iPSC-derived motor neuron cultures to determine whether or not these effects are also observed in human neurons (differentiation characterized in Additional file 9). The motor neuron cultures contained 90.5 ± 1.4 % SMI32-positive cells and 88.8 ± 1.4 % Islet 1-positive cells, with large cell bodies, consistent with a large proportion of the cells having a motor neuron morphology (Additional file 10). Treatment of human motor neuron cultures with aggregated SOD1 promoted membrane perturbations, such as ruffling and blebbing (Fig. 6 arrowheads). This membrane perturbation, quantified with the membrane dye FM1-43, was significantly increased compared to cells treated with PBS alone and similar to that induced by PMA (Fig. 7a-b, Additional file 11B). Furthermore, human neurons treated with SOD1 aggregates exhibited an increase in fluid phase uptake (Fig. 7c-d, Additional file 11A). Lastly, as observed for NSC-34 cells, the uptake of SOD1 aggregates into neurons was suppressed by the Na^+^/H^+^ exchanger inhibitor, EIPA (Fig. 7e, Additional file 11C), the inhibitor of PKC, rottlerin (Fig. 7f, Additional file 11C) and the Rac1 inhibitor, W56 (Fig. 7g, Additional file 11C).

### Activation of membrane ruffling is not restricted to SOD1 aggregates

**Fig. 8:**
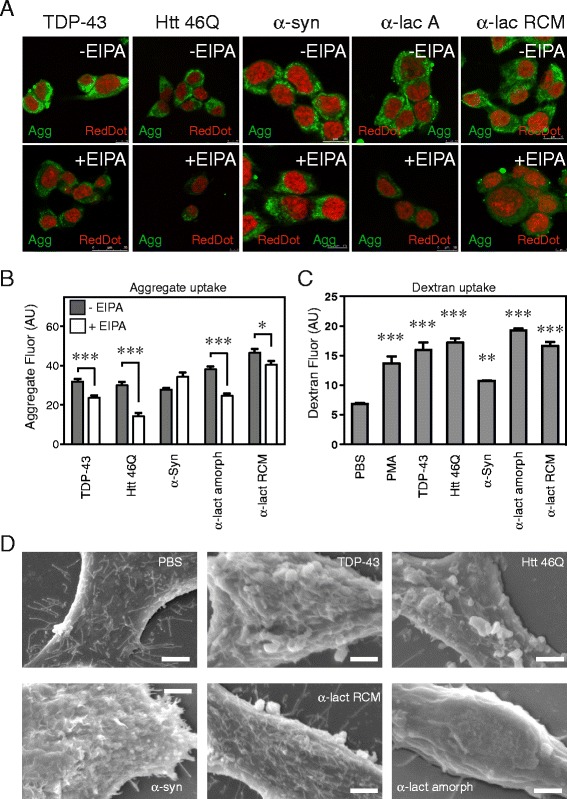
Other aggregated proteins are capable of stimulating plasma membrane ruffling (**a**) Laser scanning confocal micrographs of uptake of TDP-43, Htt_ex1_46Q, α-synuclein, and α-lactalbumin aggregates into NSC-34 cells. Cells were incubated with the aggregated proteins (20 μg/mL) in the presence or absence of EIPA. Fixed and permealized cells were labelled with an ALEXA-488 conjugated to SA (green) and RedDot 2 (red). (**b**) ImageJ quantification of SOD1 uptake in to treated NSC-34 cells. Results shown as mean fluorescence intensity per cell, minimum 50 cells, ± SD, ** *p* < 0.01. (**c**) Flow cytometry of NSC-34 cells co-treated with protein aggregates and dextran-Alexa647. Results shown as means ± SD, *n* =3, *** P <0.001. (**d**) Field emission SEM of cells treated with protein aggregates

Given that SOD1 aggregates were found to trigger the activation of membrane ruffling and entry via macropinocytosis, we sought to determine if triggering of membrane ruffling was responsible for a generic cellular response to aggregates. We therefore examined the uptake of disease-associated fibrillar aggregates formed by TDP-43, Htt_ex1_-46Q, α-synuclein, and also of amorphous and fibrillar aggregates formed from the model protein α-lactalbumin [38] into NSC-34 cells (Fig. 8, Additional file 12). Using laser scanning confocal microscopy we confirmed the uptake of aggregates in each case (Fig. 8a) and, with the exception of α-synuclein, the levels of uptake were significantly suppressed by EIPA (Fig. 8a-b). Moreover, all aggregates induced a significant (*p* < 0.01) increase in the uptake of the fluid phase marker dextran-Alexa647 (Fig. 8c) and an increase in membrane perturbations, as illustrated in FESEM images (Fig. 8d). Thus, a broad range of aggregated proteins, both amorphous and amyloid-like, are able to induce membrane perturbations that facilitate their cellular uptake in a similar manner to that of SOD1 (Fig. 8).

## Discussion

The work presented here reveals that both soluble and aggregated SOD1 are taken up by neuronal cells via fluid phase endocytosis pathways. In addition we show that aggregated but not soluble SOD1 triggers activation of Rac1 and subsequent membrane ruffling, and thus itself stimulates macropinocytosis, not only in cell lines but also in human iPSC derived motor neurons. This process differs from that responsible for the uptake of soluble SOD1, which is independent of membrane ruffling and occurs presumably via a constitutive form of pinocytosis in the case of the wtSOD1 and potentially via a range of mechanisms in the case of soluble G93A SOD1. SOD1 aggregates are not permanently maintained in a membrane bound compartment once inside the cells, but rupture macropinosomes and escape into the cytosol where they can induce further aggregation. In addition, the data presented here shows that other protein aggregates, including those of TDP-43, Htt_ex1_-46Q, α-synuclein, and α-lactalbumin, can also trigger significant perturbations in the plasma membrane of neurons allowing the uptake of large fibrillar or amorphous protein aggregates.

Various viruses, such as the vaccinia virus, adenovirus 3, herpes simplex virus 1 and HIV, utilize macropinocytosis to gain entry to cells. This phenomenon is likely to be due to the fact that macropinosomes are not restricted in size, enabling even large virions to be internalized, and that many cell types, not just professional phagocytes (such as macrophages), have the ability to activate the macropinocytosis pathways [18]. Indeed, macropinocytosis can be activated in neurons by interactions with large viral particles [21]. In the specific context of ALS we show that aggregates formed by SOD1 can activate the same macropinocytosis pathways as those utilized by virions and that this can result in the propagation of aggregation. In addition, we show that aggregates formed from TDP-43, which are also associated with ALS, stimulate ruffling and mediates their subsequent cellular uptake. Previous work has shown that amyloid fibrils can enhance HIV viral infection [39], it is interesting to speculate that this behaviour could be due to the potent ability of such aggregates to stimulate macropinocytosis.

Our data are consistent with the notion that protein aggregates could be an active part of ALS disease progression, possibly through secondary nucleation [40], or another prion-like aggregate propagation process. Recent work provides striking evidence that this may indeed drive disease progression as focal injection of spinal cord homogenates from symptomatic G93A SOD1 triggers progressive motor neuron disease in mice expressing G85R SOD1-YFP below the threshold for disease [15]. Our data however, does not directly demonstrate seeding of aggregation and as such we cannot rule out the possibility that the stress of aggregate uptake induces further aggregation in the absence of seeding. We have also shown that Rac1 and protein kinase C (PKC) pathways are actively involved in aggregate uptake. Interestingly, intracellular mutant SOD1 has been found to interact with and activate Rac1 [41]. Rac1, a Rho GTPase, has been identified as an important and central player in triggering membrane ruffles associated with virus entry into cells and has been found to do so by activating downstream effectors of actin polymerisation [42]. In addition, while the precise role of PKC in virus entry is still unclear, its activation with PMA (as used in this study) can induce ruffling and fluid uptake in the absence of ligands that bind the cell surface [43].

There is a growing wave of evidence that is consistent with the hypothesis that protein aggregates propagate between cells and that this is responsible for the orderly progression of disease pathology seen in some neurological disorders [44–49]. The most startling evidence shows that healthy cells grafted into the brains of people with Parkinson’s disease acquires intracellular inclusions of α-synuclein, known as Lewy bodies [47]. Evidence suggests that large fibrillar aggregates of a range of proteins (tau, α-synuclein, and expanded polyglutamine repeats) are able to gain access to the cytoplasmic compartment via an incompletely understood mechanism and induce protein aggregation [44, 45, 48, 49]. We show here that aggregates of proteins associated with ALS, Parkinson’s and Huntington’s disease all trigger both membrane ruffling and fluid uptake; findings indicating the stimulation of macropinocytosis.

While much of the focus on toxic aggregate species has been placed on oligomers, our data adds to work already published that demonstrates large insoluble aggregates can be taken up by cells and subsequently promote propagation of aggregation [16, 23, 44–49]. It has previously been shown that aggregation of α-synuclein can be triggered in an acidic environment and that this, through secondary nucleation, may produce a range of aggregates including toxic oligomers [40]. Taken together, these data suggest that the propagation of large insoluble aggregates through cellular uptake may be responsible for the perpetual propagation and generation of toxic oligomeric species. This conclusion, together with the knowledge of intracellular pathways that result in macropinosome formation and closure, provide possible therapeutic targets for halting the propagation of aggregation in these disorders. For example, we have previously shown that microglia recognize protein aggregates through CD14, scavenger and toll-like receptors, and hence may use receptor-mediated endocytosis, rather than macropinocytosis, to facilitate the uptake of aggregates and to promote their degradation [50, 51]. This distinction between microglia and neurons provides a possible approach to specifically block the uptake of aggregates by neurons.

## Materials and methods

### Cell culture

The mouse neuroblastoma x spinal cord hybrid cell line (NSC-34 cells [52]) were routinely cultured in DMEM/F12 supplemented with 10 % (v/v) FBS and 2 mM GlutaMAX. Cells were maintained in an incubator at 37 °C under a humidified atmosphere containing 5 % (v/v) CO_2_. Human fibroblasts were sourced from non-ALS individuals (female aged 62 and male aged 59 at the time of collection) and reprogrammed into induced pluripotent stem cells using mRNA (Miltenyi). Pluripotency was confirmed by PluriTest and differentiation of the cells [53]. Karyotyping was carried out in iPSCs, to ensure chromosomal abnormalities were not introduced during reprogramming and culture. Immunocytochemistry confirmed the expression of the pluripotency marker Oct4 (Additional file 9A).

Differentiation of pluripotent stem cells into motor neurons was carried out as in [54] and one clone from each line was used in experiments. Motor neurons (150,000 cells) were plated onto laminin (20 μg/mL) and fibronectin (10 μg/mL) coated 13 mm coverslips. The timeline summarizes the differentiation stages and the growth factor conditions used during differentiation (Additional file 9B). The morphological changes of each cell line were examined at each stage of the differentiation process (Additional file 9B).

Confirmation of motor neuron phenotype was carried out, including expression analysis by quantitative reverse transcription PCR and immunocytochemistry. The differentiated neurons expressed the motor neuron specific markers SMI32 and islet 1 (Additional file 9C). Immunocytochemistry identified the presence of extended dendrites ~100 μm in length. Quantitative reverse transcription PCR analysis identified the expression of the motor neuron specific gene *MNX1* (that encodes the transcription factor homeobox 9, HB9) [55]. *MNX1* was specifically expressed in motor neurons and *MNX1* was silent in pluripotent stem cells. The cholinergic specific marker acetylcholine esterase (*ACHE* that encodes the enzyme responsible for the degradation of the neurotransmitter acetylcholine) was specifically expressed in cholinergic motor neurons. The expression levels for both *MNX1* and *ACHE*, were normalized to the housekeeper gene *GAPDH* (Additional file 8D).

### Application of aggregates to cells

Wt and G93A SOD1 were expressed and purified from *E.coli* as previously outlined [50, 56]. SOD1 aggregation was performed in vitro as previously described [50]. Briefly, solutions of purified wt or G93A mutant SOD1 protein (1 mg/mL) in PBS were co-incubated with 20 mM dithiothreitol (DTT) and 5 mM ethylenediaminetetraacetic acid (EDTA) for 72 h at 37 °C with shaking; aggregated SOD1 was washed several times to remove DTT and EDTA. NSC-34 cells were cultured in 12 well plates and were transfected with wt or mutant SOD1-GFP using lipofectamine 2000 (following the manufacturer’s instructions). Lipofectamine was removed after 5 h and replaced with 10 % FCS in DMEM. After 24 h the aggregates, or soluble (non-aggregated) wtSOD1 as a control, were added in fresh media to transfected or naïve NSC-34 cells. Cells were incubated for a further 48 h and then imaged. In other experiments, aggregates were added to untransfected NSC-34 cells and incubated for various time periods in the presence or absence of pathway inhibitors before fixation and detection of aggregates (see online methods for details). In some experiments, NSC34 cells were incubated with 20 μg/mL of human wt and mutant SOD1 aggregates for 1 h at 37 °C. Post incubation, cells were washed three times in PBS and incubated with trypsin (0.25 %, Invitrogen) for 5 min to remove surface-bound aggregates. The resulting detached cells were centrifuged at 1100 × g for 5 min, re-plated in media, and allowed to recover for 6 h at 37 °C before fixation for immunocytochemistry.

### Aggregation and biotinylation of wt and G93A SOD1 aggregates

SOD1 aggregation was performed in vitro as previously described 50. Aggregated SOD1 was labelled with biotinamidohexanoic acid 3-sulfo-N-hydroxysuccinimide ester sodium salt in DMSO for 2 h at RT. The unconjugated biotin was then separated by centrifugation (21 000 x *g* for 30 min) and washed three times with PBS. The purified aggregates were then resuspended in PBS (pH 7.4). A bicinchoninic acid protein assay was performed to determine the amount of protein in solution. Aggregated forms of other proteins were obtained by incubation under conditions previously described, Httex146Q [57] , TDP-43 [58], α-synuclein [59], and α-lactalbumin [38].

### Cell surface binding and internalization of aggregated SOD1

NSC-34 cells were initially incubated with 20 μg/mL of aggregated SOD1 for 30 min at 4 °C. Cells were then fixed with 4 % (w/v) paraformaldehyde (PFA) in PBS (pH 7.4) before immunodetection. In separate experiments, cells were incubated with 20 μg/mL of aggregated wtSOD1 for 60 min at 4 °C, fixed with 4 % (w/v) PFA in PBS (pH 7.4) and permeabilized, or not, with Triton x-100. SOD1 was then detected using anti-SOD1 antibodies. Cells were imaged using a Leica TCS SPII laser scanning confocal microscope (Heidelberg, Germany). In addition, quantitative analysis of SOD1 internalisation into NSC-34 cells was performed using flow cytometry using a BD LSRII (California, USA). Cells were incubated with 20 μg/mL of aggregated SOD1 for 60 min at 4 °C. Cells incubated with PBS (pH 7.4) alone acted as the control. Similar experiments were performed in the presence of the dye, Lysotracker Red as per the manufacturer’s instructions. In addition, some cells were treated with trypsin and washed extensively before being lysed after incubation with aggregated wtSOD1 for 60 min. The cell lysates were analysed by Western blotting.

Internalisation of aggregated SOD1 was measured in the presence or absence of a range of compounds that inhibit various internalisation mechanisms. NSC34 cells were pre-treated with various endocytic inhibitors including 100 μM 5-N-ethyl-N-isopropyl-amiloride (EIPA), 5 μM chlorpromazine hydrochloride (CPZ), 10 μM genistein (Gen) or 3 μM rottlerin (Rot) diluted in 1 % BSA/PBS for 30 min at 37 °C, followed by 20 μg/mL aggregated wtSOD1 for 30 min at 37 °C. Cells were then fixed and permeabilized before detection of biotinylated aggregates with SA- Alexa 488. Cells were washed once with PBS medium and analysed using a LSR II flow cytometer (BD Biosciences, San Diego, CA) (excitation 488 nm, emission collected with 515 ± 20 band-pass filters). The mean fluorescence intensity (MFI) of relative SOD1 uptake was determined using FlowJo software (Tree Star, Ashland, OR). For confocal microscopy, cells remained on glass coverslips and incubated in wells as outlined for flow cytometry. Sytox Red (5 nM) was used as a counter stain. Results are the average of at least five independent experiments.

### Field emission scanning electron microscopy (FESEM)

NSC-34 cells in phenol-red serum free culture medium were plated into 12-well plates with 19 mm glass coverslips (7x10^4^ cells/ ml/well) and starved of serum for 24 h, and treated with 20 μg/mL soluble or aggregated proteins in PBS or PBS containing 200 nM PMA for 2 h at 37 °C. Post incubation, cells were washed three times in PBS then fixed in 2.5 % glutaraldehyde/ 4 % PFA in 0.1 M phosphate buffer (pH 7.4) for 3 h at 4 °C. The cells were then washed three times in phosphate buffer and postfixed in 2 % OsO4/ water at RT for 1 h. After washing with water, the cells were dehydrated using a gradient of ethanol at 30, 50, 70, 80, 90 and 100 % (30 min per incubation) at RT. The cells were then critical point dried for 2 h using a LEICA CPD030 (Vienna, Austria) and coated with graphite-gold in a sputter coater. The samples were analysed with a JEOL 6490LV SEM (Tokyo, Japan) operated at 10 kV at a 10 mm working distance and a spot size setting of 35.

### Rac1 activation assays

NSC-34 cells were treated with 20 μg/mL of soluble and aggregated G93A SOD1 for 30 min at 37 °C. The cells were washed twice with cold PBS and harvested by treatment with 0.05 % trypsin for 10 min at 37 °C. Rac1 activation was measured using a G-LISA activation kit (Kit #BK128 Cytoskeleton, Inc. (Denver, USA) as per the manufacturer’s recommendations.

### Transmission electron microscopy

Negative staining was performed using substrate carbon-coated nickel grids (Proscitech Kirwan, Australia). Protein was loaded onto the grid and washed three times with milli-Q water. Subsequently, 2 % (w/v) uranyl acetate (ProsciTech Kirwan, Australia) in 0.22 μm sterile filtered milli Q water was added for 2 min to stain the proteins negatively. The grids were analysed using a JEOL 2011 TEM (Tokyo, Japan) operated at 200 kV and Images taken using a Gatan Orius digital camera (California, USA).

### Pharmacological inhibitors and antibodies

Pharmacological inhibitors were prepared in either DMSO or 20 % acetonitrile/water according to the manufacturer’s recommendations and used at the indicated concentrations. EIPA, CPZ, Gen, and Rot were purchased from Sigma Aldrich. The Rac1 inhibitor W56 was purchased from Tocris Bioscience.

Specific antibodies including mouse anti-beta actin (ab8226), rabbit anti-EEA1 antibody (ab2900), Anti-LAMP1 [H4A3], anti-beta actin antibody [AC-15], anti-beta tubulin antibody, anti-neuron specific beta III tubulin were purchased from Abcam. Alexa Fluor 488 goat anti-mouse, Alexa Fluor 488 goat anti-rabbit, streptavidin Alexa Fluor 633 conjugate, streptavidin Alexa Fluor 488 conjugate, Alexa Fluor 488 donkey anti-sheep, Alexa Fluor 488 donkey anti-rabbit, SYTOX Red dead cell stain, FM® 1-43FX fixable analogue of FM® 1–43 membrane stain were purchased from Invitrogen Life Technologies. Donkey anti-sheep/goat IgG HRP conjugate and goat anti-mouse IgM + IgG + IgA (H + L) HRP conjugates were purchased from Millipore.

Sheep anti-SOD1 was purchased from Thermo Fisher Scientific. Mouse monoclonal anti-human TARDBP antibody (clone k1B8) was purchased from Abnova. Anti-BiP/GRP78 was purchased from BD Transduction Laboratories. FITC-conjugated sheep anti-mouse was purchased from Silenus. RedDot 2 was obtained from Biotium. Goat Anti-Rabbit IgG (H + L)-HRP Conjugate was obtained from Bio-Rad.

### Selective permeability of cells

NSC-34 cells were incubated with 20 μg/mL of biotinylated aggregated wt and G93A SOD1 in PBS for either 60 or 120 min at 37 °C. Post incubation, cells were fixed and permeabilized with either 10 μM digitonin or 0.5 % Triton-x100 (v/v) for either 10 or 30 min at 4 °C respectively. The cells were washed three times in PBS, blocked in 5 % BSA/PBS, for subsequent detection using SA-Alexa 633. Cells were visualised using a TCS SP laser scanning confocal microscopy (Leica Microsystems, Wetzlar, Germany) using a 60x objective. The He Ne laser (633 nm) was used and emission was collected at 645 +/− 20 nm using a standard PMT. Data were acquired in Leica Application Suite (Leica Microsystems).

### Cellular subfractionation

NSC-34 cells were incubated with 20 μg/mL of aggregated SOD1 in PBS for 120 min at 37 °C. Post incubation the cytosolic (CEB), membrane (MEB), nuclear (NEB) and cytoskeletal proteins (PEB) were extracted from NSC-34 cells using a Subcellular Protein Fractionation Kit for Cultured Cells (Thermo Fisher Scientific) according to the manufacturer’s instructions. Aliquots of cell extract (20 μg protein/lane) were separated under reducing conditions (5 % β-mercaptoethanol) using discontinuous TGX Stain-Free™ Precast Gels separating gels (BioRad). Proteins were then transferred to nitrocellulose membranes (Bio-Rad, Hercules, CA) then blocked with heat denatured casein (HDC) in PBS (pH 7.4) for 1 h at 37 °C. To detect exogenously applied SOD1, sheep polyclonal anti-human SOD1 was used. To test the quality of the fractionation, rabbit anti-EEA1, anti- vimentin and mouse anti- actin antibody diluted in HDC/PBS for 1 h at 37 °C were used to probe the MEB and PEB fractions. Membranes were visualised using chemiluminescent substrate and Amersham Hyperfilm ECL (GE Healthcare, Little Chalfont, Bukinghamshire, UK). Images of films were collected using a GS-800 Calibrated Densitometer (Bio-Rad).

### Membrane dye uptake

NSC-34 cells were treated with 20 μg/mL of soluble or aggregated wt or G93A SOD1, PBS alone, or a positive control containing 200 nM PMA in PBS for 2 h at 37 °C. The cells were then washed twice in PBS and incubated with 10 μM of FM® 1-43FX membrane stain in PBS for 7 min at 37 °C. Excess dye was removed by several washes in PBS and cells were returned to the incubator for 4 min. This procedure was repeated to give a total of 8 min of incubation in PBS. Post incubation, ice-cold PBS was added to stop endocytosis and prepare cells for fixation in 4 % (w/v) PFA/PBS (pH 7.4) for 20 min at 4 °C. Post fixation, cells were washed twice in PBS and incubated with 1x RedDot 2 for 10 min at RT.

### Fluid phase uptake assays

Pinocytosis involves uptake of solutes from the extracellular medium. One well established solute is dextran. To quantify the amount of fluid phase solute uptake, NSC34 cells were treated with either 20 μg/mL of soluble and biotinylated aggregated wt and G93A SOD1, Htt_ex1_46Q, α-synuclein, TDP-43, and α-lactalbumin in PBS alone or containing 200 nM PMA for 30 min at 37 °C. Prior to harvesting or fixation, cells were incubated for 15 min with 0.5 mg/ml 10 kDa 647-dextran (Invitrogen) at 37 °C. The cells were then placed on ice to stop dextran uptake and cells were washed three times with ice cold PBS and once with low pH buffer (0.1 M sodium acetate, 0.05 M NaCl, pH 5.5) for 10 min. The cells were then prepared for either flow cytometry or confocal laser scanning microscopy as described above. For flow cytometry, dextran uptake was displayed as fluorescence mean of three or more independent experiments.

### Fixed cell antibody staining of iPSCs

The iPSCs were plated on matrigel-coated 8 mm coverslips at a density of 25,000 cells and cultured for 3 days before staining. Cells were fixed with 4 % (v/v) PFA at room temperature for 10 min, permeabilized with 0.5 % (v/v) TritionX-100 in phosphate buffered saline (PBS) and blocked with 5 % (w/v) bovine serum albumin (BSA) in PBS.

The iPSC colonies were stained with Oct3/4 (mouse 1:500) (Stem Cell Technologies) primary antibody overnight at 4C and anti-mouse Alexa Fluor 488 (1:1000) (Life Technologies) secondary antibody for 1 h at room temperature.

### Fixed cell antibody staining of motor neurons

Cells were plated on coverslips coated with laminin (20 μg/mL) and fibronectin (10 μg/mL) at a density of 42,000 cells/cm^2^. Cells were fixed using 4 % (v/v) PFA at room temperature for 10 min. The cells were permeabilized with 0.5 % (v/v) Triton X-100 in PBS at room temperature for 15 min. Cells were blocked with 5 % (w/v) bovine serum albumin (BSA) in PBS at room temperature for 1 h. SMI32 primary antibody (Abcam) was diluted 1:800 in PBS 5 % BSA and incubated at 4 °C overnight. Secondary antibodies, Alexa Fluor 488 anti-sheep IgG antibody (1:1000 in PBS 5 % BSA) was incubated with the cells for 1 h at room temperature. Images of stained cells were taken on a Leica DMI6000B confocal microscope and acquired using the LAS AF 2.3.5 software.

### Quantitative RT-PCR

RNA was extracted and purified from differentiated cell using the ISOLATE II RNA Mini Kit (Bioline, USA), as per manufacturer’s instructions. The purified RNA was quantified using a Nanodrop 2000C (Thermo Fisher Scientific, USA).

RNA was reverse transcribed into complementary DNA (cDNA) for subsequent analysis. Reagents for cDNA preparation were obtained from Promega (USA). Five μg of purified RNA was annealed to random primers (0.75 μg) and oligo dT primers (0.75 μg) by incubating at 65 °C for 4 min, followed by 1 min incubation on ice. For reverse transcription, Moloney-murine leukaemia virus reverse transcriptase (M-MLV RTase) (150 U), 96 nmol dNTPs, RNasin (60 U) and 1x MMLV RTase Buffer were added to the reaction mixture and then incubated at 37 °C for 100 min.

The primers for qRT-PCR were obtained from Sigma Aldrich (USA) (unless stated otherwise) and had the following sequences:

- acetylcholinesterase (AChE)
- forward: 5’-GGAACCGCTTCCTCCCCAAATTG-3’,
- reverse: 5’-TGCTGTAGTGGTCGAACTGGTTCTTC-3’; Homeobox 9 (MNX1)
- forward: 5’-GTTCAAGCTCAACAAGTACC-3’,
- reverse: 5’-GGTTCTGGAACCAAATCTTC-3’; GFAP
- forward: 5’-CTGGATCTGGAGAGGAAGATTGAGTCG-3’,
- reverse: 5’-CTCATACTGCGTGCGGATCTCTTTCA-3’;  glyceraldehyde 3-phosphate dehydrogenase (GAPDH) forward: 5’-GAGCACAAGAGGAAGAGAGAGACCC-3’, reverse: 5’-GTTGAGCACAGGGTACTTTATTGATGGTACATG-3’. The final qRT-PCR reaction consisted of 10 μL of SYBR Select Master Mix, 800 nM of each forward and reverse primer, 2 μL of cDNA in a final reaction volume of 20 μL. Each reaction was run in duplicate and a negative control (water) and no reverse transcription (RNA) control was included as well as a positive control using cDNA of human putamen. The amplification consisted of 40 cycles, of 95 °C for 15 s (activation step), 58 °C for 15 s (annealing step) and 72 °C for 1 min. A melting curve analysis was conducted to confirm the presence of the appropriate amplified target. The acquired data was normalized against quantitative expression levels of the housekeeping gene GAPDH and analyzed using the comparative threshold cycle method.

### Preparation of giant unilamellar vesicles

The rapid evaporation method was used to prepare giant unilamellar vesicles for confocal microscopy as described in [36]. Briefly, soy L-α-phosphatidylcholine (Avanti Polar Lipids Inc) was dissolved in CHCl_3_:MeOH (2:1) to give a phospholipid concentration of 5 mM. Liposome buffer (50 mM HEPES, 107 mM NaCl, 1 mM EDTA, 0.1 M sucrose, pH 7.4, 2.5 mL) was then added to the lipid/solvent solution in a 50 mL round bottom flask and the two phases mixed by vigorous pipetting. The organic solvent was removed by rotary evaporator under reduced pressure (final pressure 44 mbar) for 5 min at 35 °C. The resulting liposome suspension was stored overnight at 4 °C prior to confocal microscopy studies.

## Additional files

Additional file 1:
**SOD1 aggregates in to fibril like structures that associate with cells via membrane proteins.** (PDF 1780 kb) Additional file 2:
**SOD1 aggregates are internalized in NSC-34 cells.** (PDF 52 kb) Additional file 3:
**Aggregates made from a variety of SOD1 mutants induce formation of wtSOD1-GFP aggregates.** (PDF 471 kb) Additional file 4:
**Aggregated and soluble SOD1 enter the cytosol of NSC-34 cells.** (PDF 3626 kb) Additional file 5:
**Disruption of membrane structure by SOD1 aggregates.** (PDF 1099 kb) Additional file 6:
**Small molecule inhibitors block macropinocytosis.** (PDF 253 kb) Additional file 7:
**Addition of SOD1 does not induce rapid apoptosis.** (PDF 89 kb) Additional file 8:
**Rac1 activation is downstream of membrane ruffling.** (PDF 52 kb) Additional file 9:
**Characterization of iPSC derived motor neurons.** (PDF 6958 kb) Additional file 10:
**Characterization of iPSC derived motor neurons.** (PDF 1487 kb) Additional file 11:
**wtSOD1 aggregates activate membrane perturbation and dextran uptake in iPSC derived human motor neurons.** (PDF 71 kb) Additional file 12:
**Morphology of protein aggregates using TEM.** (PDF 3960 kb)
