# Supplementary material for: SOD1 protein aggregates stimulate macropinocytosis in neurons to facilitate their propagation
Source: Mol Neurodegener. 2015 Oct 31;10:57. doi: 10.1186/s13024-015-0053-4 (PMC4628302; doi:10.1186/s13024-015-0053-4)
Supplement: Additional file 1: — SOD1 aggregates in to fibril like structures that associate with cells via membrane proteins. (PDF 1780 kb) [file 13024_2015_53_MOESM1_ESM.pdf]

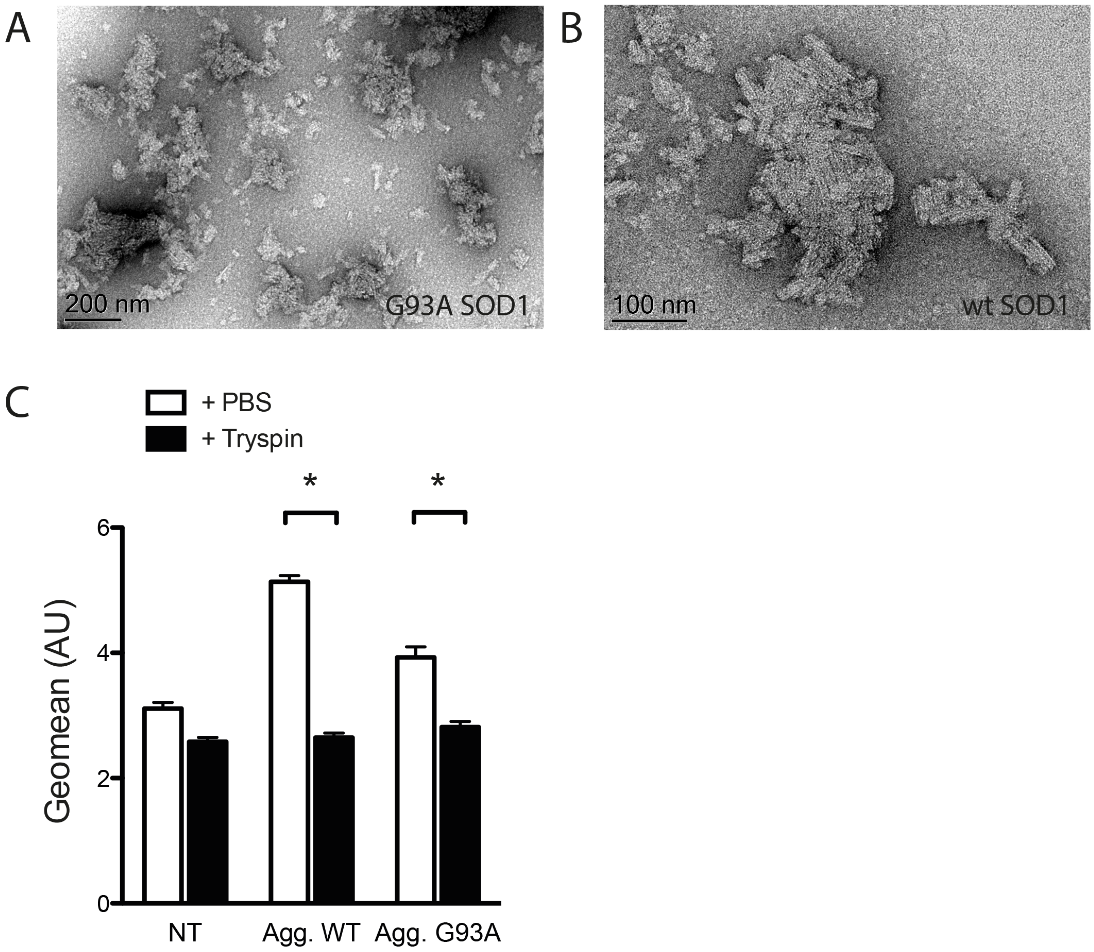

Additional File 1. SOD1 aggregates in to fibril like structures that associate with cells via membrane proteins. (A-B) Aggregation of purified wt and G93A SOD1. Representative TEM image of endpoint aggregates, scale bar are as indicated. (C) Internalization of aggregated wt and G93A SOD1 (20 µg/mL) proteins for 30 min at 37°C, in the absence (control) or presence of a pre-incubation step with 0.05% trypsin for 10 min at 37°C. MFI of Alexa647-dextran uptake was measured using flow cytometry. Results shown as geometric means ± SD, n =6, * P <0.05.
