# Supplementary material for: SOD1 protein aggregates stimulate macropinocytosis in neurons to facilitate their propagation
Source: Mol Neurodegener. 2015 Oct 31;10:57. doi: 10.1186/s13024-015-0053-4 (PMC4628302; doi:10.1186/s13024-015-0053-4)
Supplement: Additional file 2: — SOD1 aggregates are internalized in NSC-34 cells. (PDF 52 kb) [file 13024_2015_53_MOESM2_ESM.pdf]

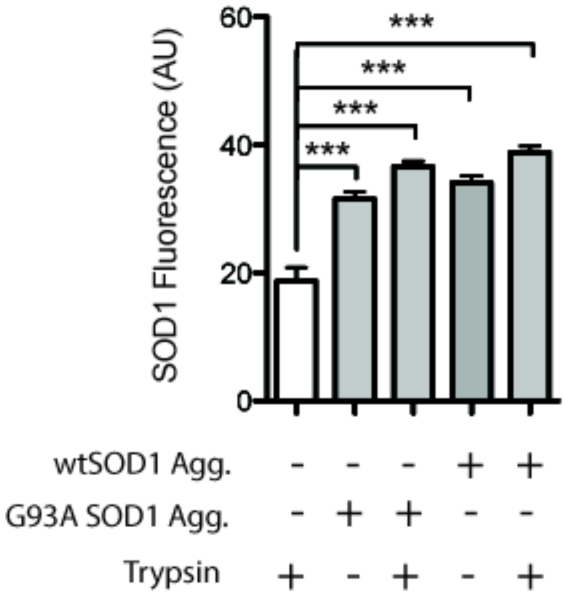

Additional File 2. SOD1 aggregates are internalized in NSC-34 cells. NSC34 Cells were incubated with 20 µg/mL of HuWt and HuG93A SOD1 aggrgates for 1 h at 37°C. Cell were incubated with trypsin (0.25%) or PBS for 5 min to remove surface-bound aggregates. The resulting detached cells were re-plated in media, and allowed to recover for 6 h at 37 °C before fixation for immunocytochemistry. Biotinylated SOD1 aggregates were detected using SA-alexa488. Results shown are mean cellular fluorescence means ± SD, n > 30, *** P <0.001.
