# Supplementary material for: SOD1 protein aggregates stimulate macropinocytosis in neurons to facilitate their propagation
Source: Mol Neurodegener. 2015 Oct 31;10:57. doi: 10.1186/s13024-015-0053-4 (PMC4628302; doi:10.1186/s13024-015-0053-4)
Supplement: Additional file 3: — Aggregates made from a variety of SOD1 mutants induce formation of wtSOD1-GFP aggregates. (PDF 471 kb) [file 13024_2015_53_MOESM3_ESM.pdf]

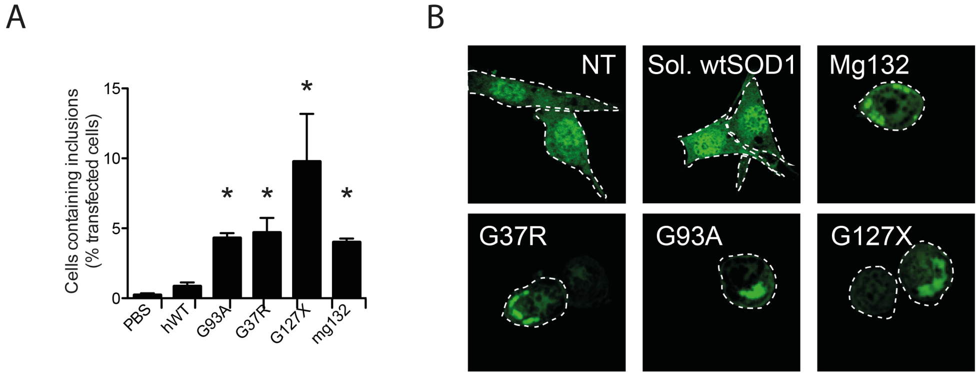

Additional File 3. Aggregates made from a variety of SOD1 mutants induce formation of wt SOD1-GFP aggregates. Cells were transfected with wt SOD1-GFP and incubated with either PBS, MG132 (10 µM for 16 hours) or mutant SOD1 variants (G93A, G37R or G127X). Cells were incubated for 48 hours before analysis for the number of inclusions. (A) The number of transfected cells that contained inclusions were counted and are reported as a percentage of all transfected cells. The data shown is calculated from triplicate experiments after counting cells from 5 random fields of view per well. Error bars represent SEM and * denotes $p<0.05$. (B) Fluorescence micrographs showing representative images of cells treated with soluble wtSOD1 or aggregated wtSOD1.
