# Supplementary material for: SOD1 protein aggregates stimulate macropinocytosis in neurons to facilitate their propagation
Source: Mol Neurodegener. 2015 Oct 31;10:57. doi: 10.1186/s13024-015-0053-4 (PMC4628302; doi:10.1186/s13024-015-0053-4)
Supplement: Additional file 4: — Aggregated and soluble SOD1 enter the cytosol of NSC-34 cells. (PDF 3626 kb) [file 13024_2015_53_MOESM4_ESM.pdf]

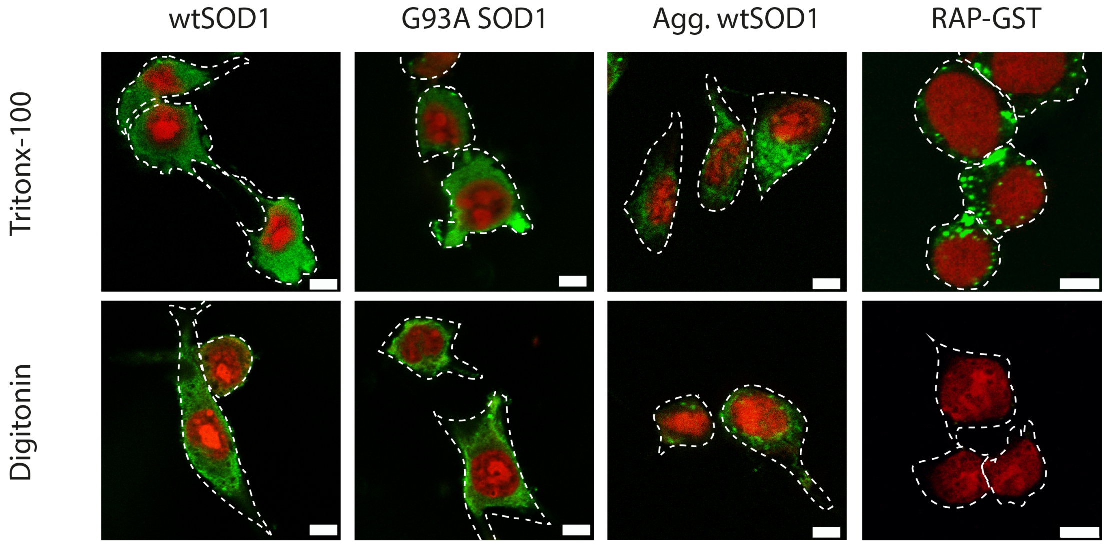

Additional File 4. Aggregated and soluble SOD1 enter the cytosol of NSC-34 cells. NSC-34 cells were treated with soluble or aggregated wt and G93A SOD1 proteins for 120 minutes. Cells were fixed and then permeabilized with Triton-x100 (0.5%) or digitonin (10 µM). Cells were labeled with Alexa-633 conjugated to SA (green; to detect biotinylated SOD1) and Red Dot 2 (red; to label nuclei). Images are laser scanning confocal microscopy. In addition, cells were treated with RAP-GST for 120 min, fixed, permeabilized (Triton-x100 or digitonin) and then stained with an anti-GST antibody. RAP-GST remained in the endomembrane system and was not detected after digitonin permeabilization.
