# Supplementary material for: SOD1 protein aggregates stimulate macropinocytosis in neurons to facilitate their propagation
Source: Mol Neurodegener. 2015 Oct 31;10:57. doi: 10.1186/s13024-015-0053-4 (PMC4628302; doi:10.1186/s13024-015-0053-4)
Supplement: Additional file 5: — Disruption of membrane structure by SOD1 aggregates. (PDF 1099 kb) [file 13024_2015_53_MOESM5_ESM.pdf]

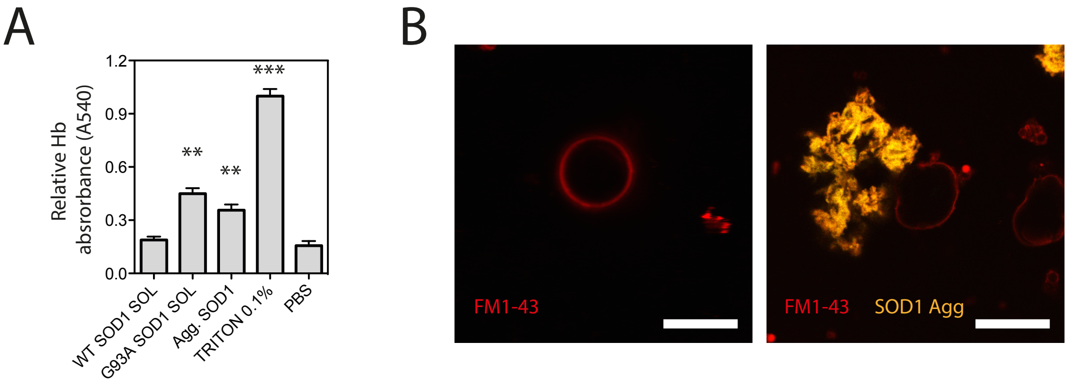

Additional File 5. Disruption of membrane structure by SOD1 aggregates. (A) SOD1 was added to human red blood cells and membrane disruption quantified as free hemoglobin (A540). Mutant SOD1 induced the most membrane damage while aggregates of wt and G93A SOD1 induced significant membrane disruption. Results shown as means ± SD, n =6, * p <0.05. (B) Unilamellar vesicles were formed from L-α-phosphatidylcholine and aggregates were added to preformed vesicles. Unilamelar vesicles were spherical in shape and contact with aggregates distorted and disrupted the vesicles.
