# Supplementary material for: SOD1 protein aggregates stimulate macropinocytosis in neurons to facilitate their propagation
Source: Mol Neurodegener. 2015 Oct 31;10:57. doi: 10.1186/s13024-015-0053-4 (PMC4628302; doi:10.1186/s13024-015-0053-4)
Supplement: Additional file 6: — Small molecule inhibitors block macropinocytosis. (PDF 253 kb) [file 13024_2015_53_MOESM6_ESM.pdf]

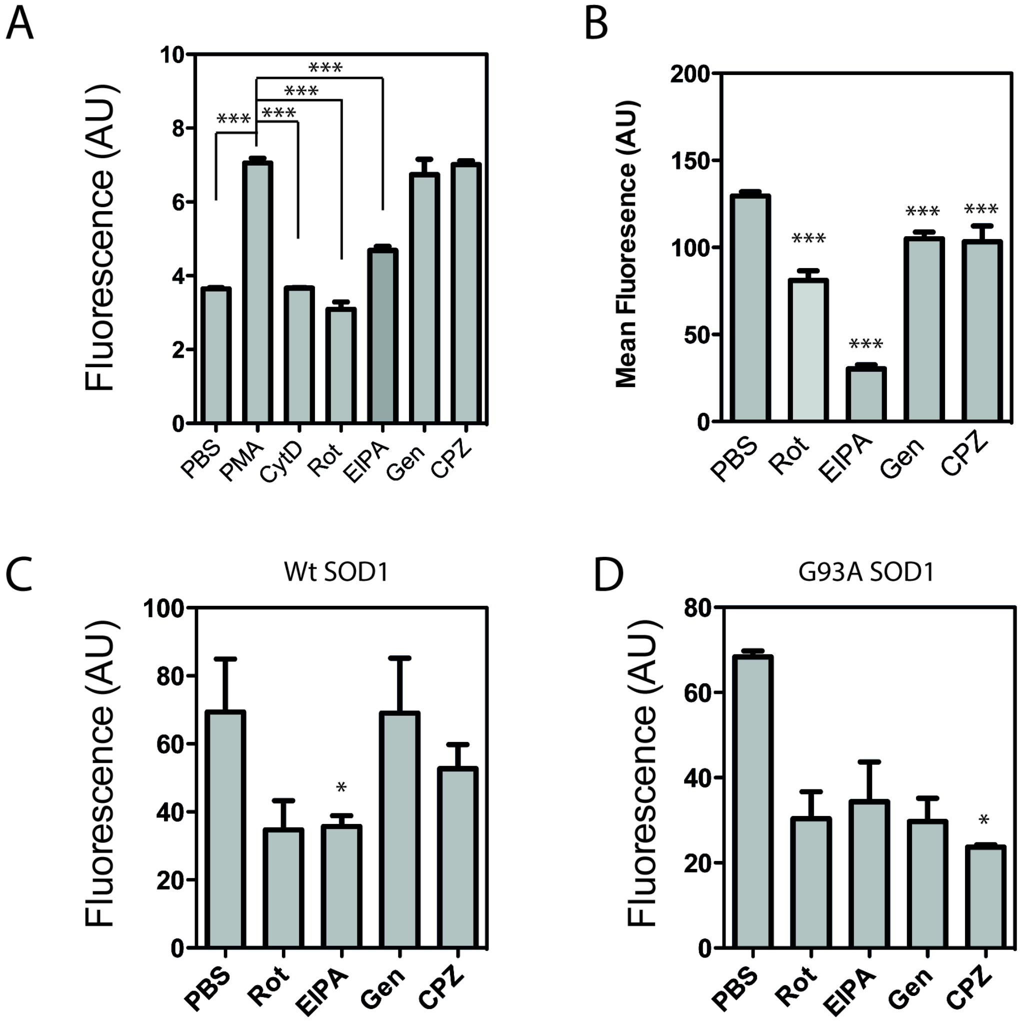

Additional File 6. Small molecule inhibitors block macropinocytosis. PMA induced dextran uptake is supressed by inhibitors of macropinocytosis (A). The induction of fluid phase uptake was measured using fluorescently labelled dextran. NSC-34 cells were serum starved for 24 h and were pre-treated with with either rottlerin (3 μM), EIPA (100 μM), genistein (10 μM) and chlorpromazine hydrochloride (CPZ) (5 mM) for 30 min at 37°C, and then co-incubated with PMA (200 nM) for an additional 30 min at 37°C. Cells were subsequently incubated with 10 kDa Alexa647-dextran (0.5 mg/ml) for 15 min prior to fixation. MFI of Alexa647-dextran uptake was measured using flow cytometry. Results shown as means ± SD, n =6, * p <0.05. Aggregated wt SOD1 uptake is inhibited by EIPA and rottlerin (B). Internalization of aggregated wt SOD1 (20 μg/mL) for 30 min at 37°C, in the absence (control) or presence of a pre-incubation step with either rottlerin (3 μM), EIPA (100 μM), genistein (10 μM) or chlorpromazine hydrochloride (CPZ) (5 mM). Fixed and permeabilized cells were labeled with Alexa488 conjugated to SA and fluorescence measured using flow cytometry. Error bars represent SD and *** denotes p<0.001. Differential inhibition of the cellular uptake of non-aggregated wt and G93A NSC-34 (C-D). Internalization of non-aggregated wt and G93A SOD1 (20 μg/mL) for 30 min at 37°C, in the absence (PBS) or presence of a pre-incubation step with either rottlerin (3 μM), EIPA (100 μM), genistein (10 μM) and chlorpromazine hydrochloride (CPZ) (5 mM). Fixed and permeabilized cells were probed with an anti-human SOD1 antibody conjugated Alexa488 and fluorescence measured using flow cytometry. Results shown as means ± SD, n = 3, * p <0.05.
