# Supplementary material for: SOD1 protein aggregates stimulate macropinocytosis in neurons to facilitate their propagation
Source: Mol Neurodegener. 2015 Oct 31;10:57. doi: 10.1186/s13024-015-0053-4 (PMC4628302; doi:10.1186/s13024-015-0053-4)
Supplement: Additional file 7: — Addition of SOD1 does not induce rapid apoptosis. (PDF 89 kb) [file 13024_2015_53_MOESM7_ESM.pdf]

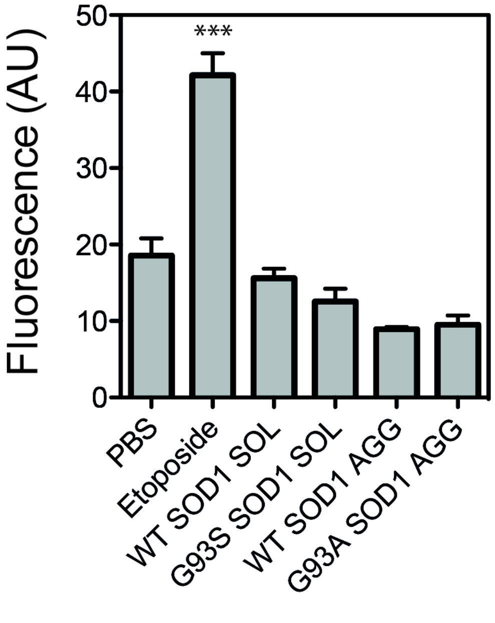

Additional File 7. Addition of SOD1 does not induce rapid apoptosis. Cells were incubated with soluble or aggregated forms of SOD1 for 2 h and then the levels of activated caspase-3 tested using Image-IT kit. While the levels of activated caspase-3 were increased in NSC-34 cells incubated overnight with etoposide, there was no increase in fluorescence of cells treated as described with any forms of SOD1. Results shown as means ± SD, n = 3, * p <0.001.
