# Supplementary material for: SOD1 protein aggregates stimulate macropinocytosis in neurons to facilitate their propagation
Source: Mol Neurodegener. 2015 Oct 31;10:57. doi: 10.1186/s13024-015-0053-4 (PMC4628302; doi:10.1186/s13024-015-0053-4)
Supplement: Additional file 8: — Rac1 activation is downstream of membrane ruffling. (PDF 52 kb) [file 13024_2015_53_MOESM8_ESM.pdf]

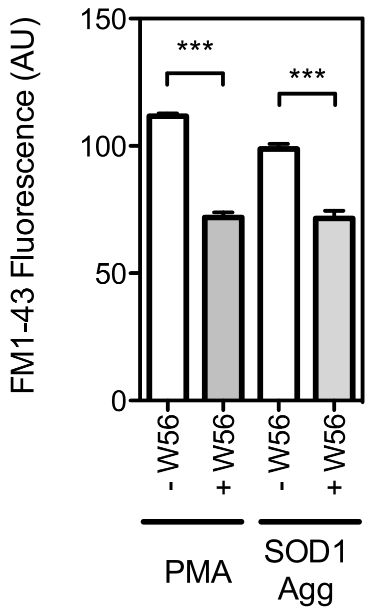

Additional File 8. Rac1 activation is downstream of membrane ruffling. Laser scanning confocal micrographs of SOD1 aggregate uptake in the presence of absence of W56 were used to calculate mean fluorescence per cell using ImageJ. NSC-34 cells were treated with PMA or SOD1 aggregates in the presence or absence of Rac1 inhibitor W56 and then examined for membrane perturbations using membrane dye FM1-43FX. Both PMA and aggregate treated cells showed reduced membrane perturbation in the presence of W56. Data are mean fluorescence intensity per cell of a minimum of 100 cells ± SD, *** $p < 0.001$.
