# Supplementary material for: SOD1 protein aggregates stimulate macropinocytosis in neurons to facilitate their propagation
Source: Mol Neurodegener. 2015 Oct 31;10:57. doi: 10.1186/s13024-015-0053-4 (PMC4628302; doi:10.1186/s13024-015-0053-4)
Supplement: Additional file 9: — Characterization of iPSC derived motor neurons. (PDF 6958 kb) [file 13024_2015_53_MOESM9_ESM.pdf]

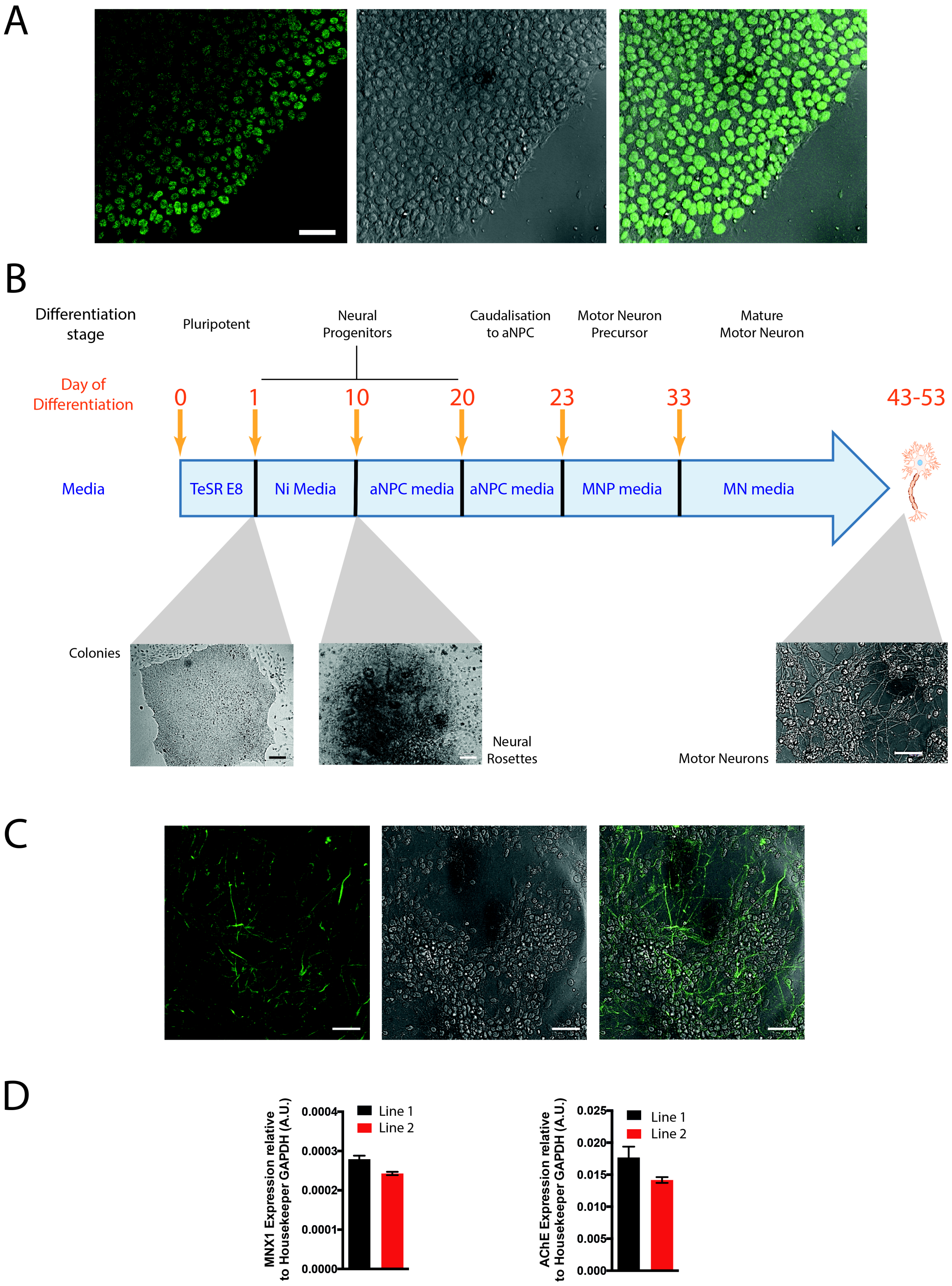
A
B
Differentiation stage
Pluripotent
Neural Progenitors
Caudalisation to aNPC
Motor Neuron Precursor
Mature Motor Neuron
Day of Differentiation
0
1
10
20
23
33
43-53
Media
TeSR E8
Ni Media
aNPC media
aNPC media
MNP media
MN media
Colonies
Neural Rosettes
Motor Neurons
C
D
MNX1 Expression relative to Housekeeper GAPDH (A.U.)
0.0004
0.0003
0.0002
0.0001
0.0000
Line 1
Line 2
AChE Expression relative to Housekeeper GAPDH (A.U.)
0.025
0.020
0.015
0.010
0.005
0.000
Line 1
Line 2

Additional File 9. Characterization of iPSC derived motor neurons. (A) Immunocytochemistry confirmed the expression of the pluripotency marker Oct4. Human induced pluripotent stem cells were cultured in TeSR-E8 on glass coverslips coated with Matrigel, fixed and stained with Alexa Fluor 488-conjugated Oct4 antibody. Confocal and brightfield images were taken on a Leica SP5 confocal microscope. Scale bar is 60 mm. (B) The timeline summarises the differentiation stages and the growth factor conditions used during differentiation (Billican et al.). Example images of cellular morphological changes during differentiation are shown. Scale bars are 100 mm, 50 mm, 50 mm respectively. (C) Motor neurons were cultured on glass coverslips coated with laminin, collagen and fibronectin. Immunocytochemistry confirmed the expression of the motor neuron marker SMI-32. Scale bars are 30 mm. (D) Expression of neuronal and cholinergic markers were quantified using quantitative RT-PCR. Data plotted as mean relative gene expression in motor neurons normalised to *GAPDH* (± SEM, n=3). *MNX1* and *ACHE* expression was silent in pluripotent cells.
