# Supplementary material for: SOD1 protein aggregates stimulate macropinocytosis in neurons to facilitate their propagation
Source: Mol Neurodegener. 2015 Oct 31;10:57. doi: 10.1186/s13024-015-0053-4 (PMC4628302; doi:10.1186/s13024-015-0053-4)
Supplement: Additional file 10: — Characterization of iPSC derived motor neurons. (PDF 1487 kb) [file 13024_2015_53_MOESM10_ESM.pdf]

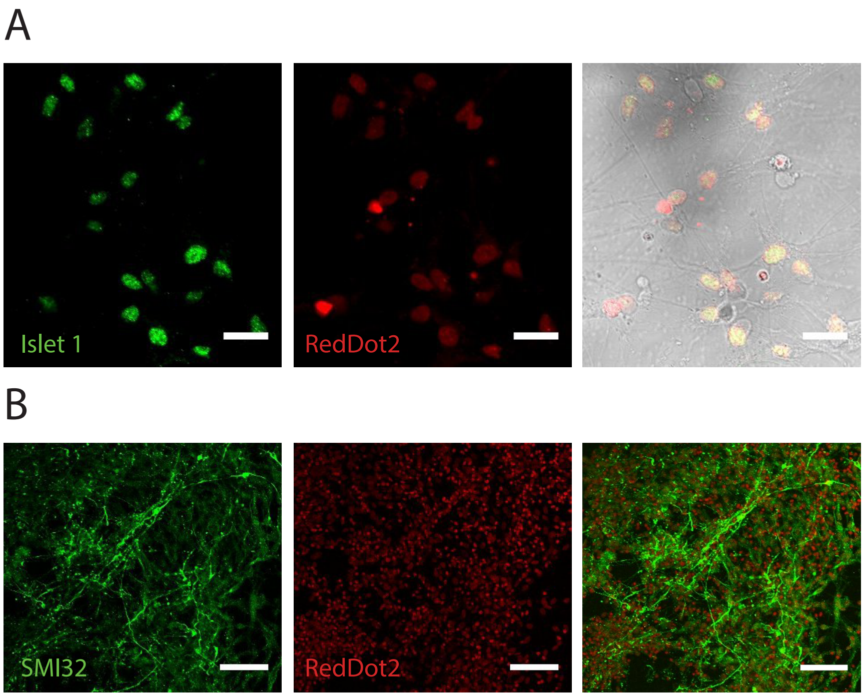

Additional File 10. Characterization of iPSC derived motor neurons. Quantification of Islet 1 (A) and SMI32 (B) positive cells was performed using immunocytochemistry and analysis using Image J software. Islet 1 and SMI32 positive cells were calculated as a percentage of the total number of cells (determined by nuclear stain) from motor neuron cultures generated from six donor cell lines, using 6-10 images from each technical replicate (separate differentiation experiments using 3 technical replicates for Islet 1 and 2 technical replicates for SMI32). The motor neuron cultures contained 90.5 ± 1.4 % SMI32-positive cells and 88.8 ± 1.4 % Islet 1-positive cells.
