# Supplementary material for: SOD1 protein aggregates stimulate macropinocytosis in neurons to facilitate their propagation
Source: Mol Neurodegener. 2015 Oct 31;10:57. doi: 10.1186/s13024-015-0053-4 (PMC4628302; doi:10.1186/s13024-015-0053-4)
Supplement: Additional file 11: — wtSOD1 aggregates activate membrane perturbation and dextran uptake in iPSC derived human motor neurons. (PDF 71 kb) [file 13024_2015_53_MOESM11_ESM.pdf]

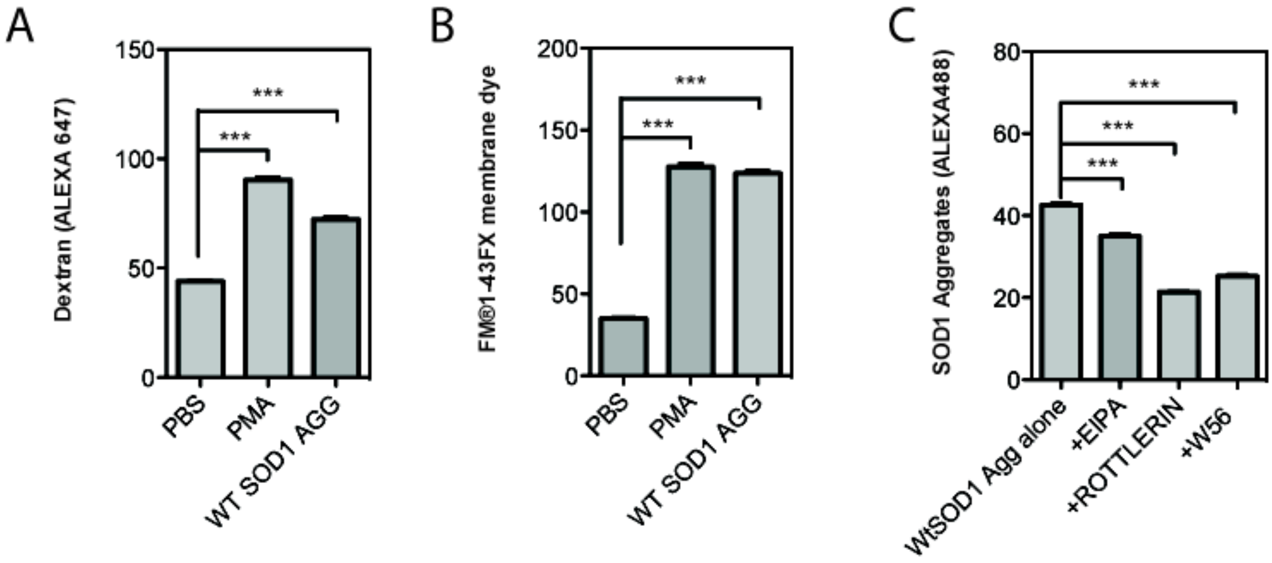

Additional File 11. wtSOD1 aggregates activate membrane perturbation and dextran uptake in iPSC derived human motor neurons. (A) The induction of fluid phase uptake was measured using fluorescently labelled dextran. Laser scanning confocal micrographs of dextran-Alexa647 uptake in treated motor neurons were quantified using ImageJ of dextran uptake in the treated neurons. A minimum of 100 cells per treatment were scored. Results shown as means ± SD of 3 experiments **** p <0.001***. Laser scanning confocal micrographs of treated cells stained with the membrane dye FM1-43FX were used to measure membrane perturbation and fluorescence intensity quantification using ImageJ. A minimum of 100 cells were scored per treatment. Results shown are means ± SD of three experiments, *** p<0.001 **. (C) Laser scanning confocal micrographs of aggregated SOD1 internalized by motor neurons in the presence or absence of a pre-incubation step with macropinocytosis inhibitors EIPA, Rottlerin and rac1 inhibitor W56 were quantified by imageJ. Data are mean fluorescence intensity per cell of a minimum of 100 cells ± SD, ***p < 0.001. Data shown is from experiments performed on cells derived from one fibroblast line and represents experiments performed on cells from 2 individuals.
