# Supplementary material for: SOD1 protein aggregates stimulate macropinocytosis in neurons to facilitate their propagation
Source: Mol Neurodegener. 2015 Oct 31;10:57. doi: 10.1186/s13024-015-0053-4 (PMC4628302; doi:10.1186/s13024-015-0053-4)
Supplement: Additional file 12: — Morphology of protein aggregates using TEM. (PDF 3960 kb) [file 13024_2015_53_MOESM12_ESM.pdf]

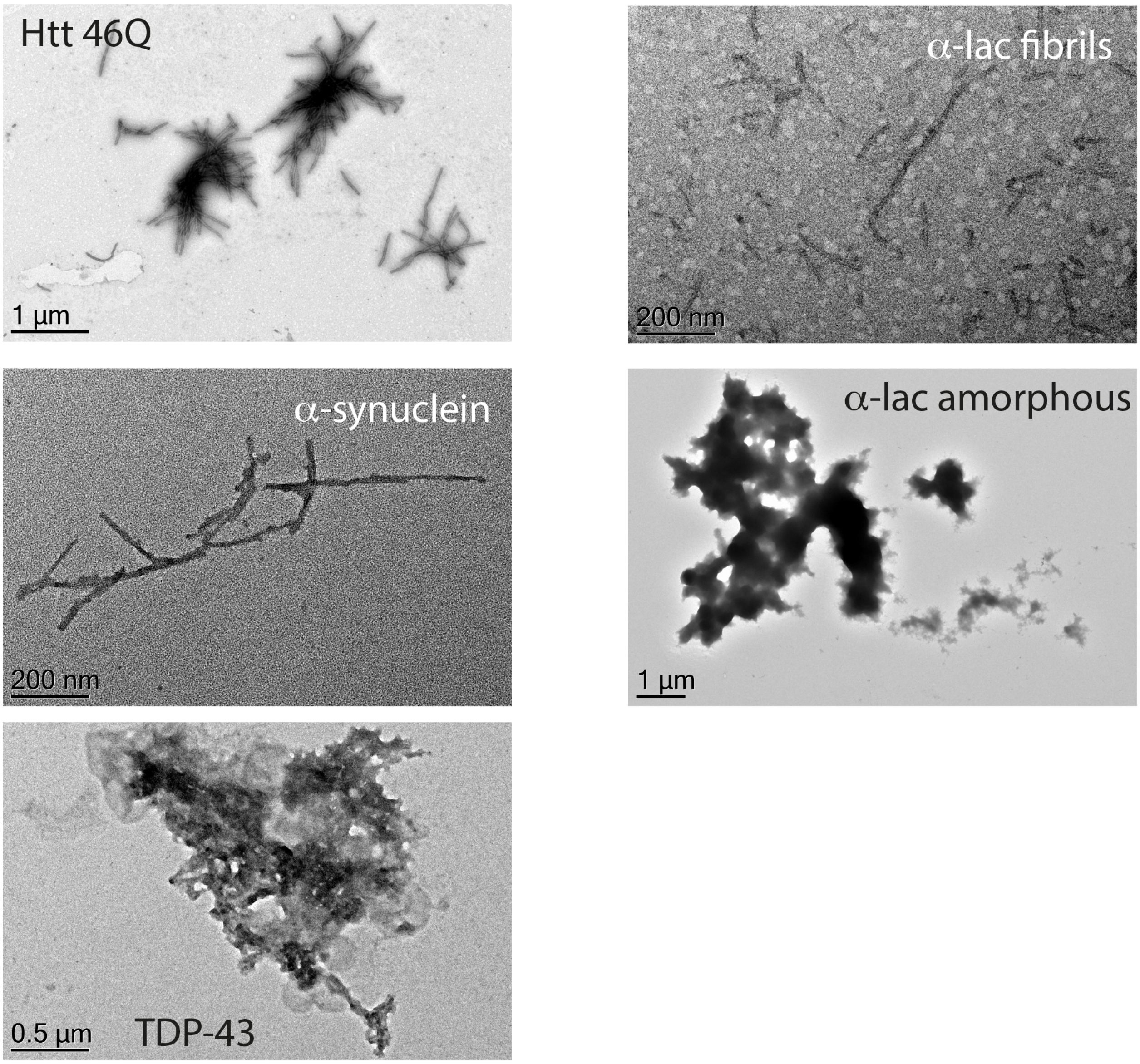

Additional File 12. Morphology of protein aggregates using TEM. Aggregation of purified TDP-43, Htt_{ex1}-46Q, α-synuclein, α -lactalbumin showing amorphous and fibrillar aggregates. Representative TEM image of endpoint aggregates negatively stained with uranyl acetate, scale bars are as indicated.
